# Review of Australian initiatives to reduce stigma towards people with complex mental illness: what exists and what works?

**DOI:** 10.1186/s13033-020-00423-1

**Published:** 2021-01-18

**Authors:** Amy J. Morgan, Judith Wright, Nicola J. Reavley

**Affiliations:** Centre for Mental Health, Melbourne School of Population and Global Health, University of Melbourne, 207 Bouverie Street, Melbourne, VIC 3010 Australia

**Keywords:** Mental illness, Stigma, Discrimination, Schizophrenia, Bipolar disorder, Psychosis, Personality disorder

## Abstract

**Background:**

Australian national mental health policy outlines the need for a nationally coordinated strategy to address stigma and discrimination, particularly towards people with complex mental illness that is poorly understood in the community. To inform implementation of this policy, this review aimed to identify and examine the effectiveness of existing Australian programs or initiatives that aim to reduce stigma and discrimination.

**Method:**

Programs were identified via a search of academic databases and grey literature, and an online survey of key stakeholder organisations. Eligible programs aimed to reduce stigma towards people with complex mental illness, defined as schizophrenia, psychosis, personality disorder, or bipolar disorder; or they focused on nonspecific ‘mental illness’ but were conducted in settings relevant to individuals with the above diagnoses, or they included the above diagnoses in program content. Key relevant data from programs identified from the literature search and survey were extracted and synthesized descriptively.

**Results:**

We identified 61 programs or initiatives currently available in Australia. These included face-to-face programs (n = 29), online resources (n = 19), awareness campaigns (n = 8), and advocacy work (n = 5). The primary target audiences for these initiatives were professionals (health or emergency), people with mental illness, family or carers of people with mental illness, and members of the general population. Most commonly, programs tended to focus on stigma towards people with non-specific mental illness rather than on particular diagnostic labels. Evidence for effectiveness was generally lacking. Face-to-face programs were the most well-evaluated, but only two used a randomised controlled trial design.

**Conclusions:**

This study identified areas of strength and weakness in current Australian practice for the reduction of stigma towards people with complex mental illness. Most programs have significant input from people with lived experience, and programs involving education and contact with a person with mental illness are a particular strength. Nevertheless, best-practice programs are not widely implemented, and we identified few programs targeting stigma for people with mental illness and their families, or for culturally and linguistically diverse communities, Aboriginal and Torres Strait Islander communities and LGBTIQ people. These can inform stakeholder consultations on effective options for a national stigma and discrimination reduction strategy.

## Background

Stigmatising attitudes towards people with mental illness are prevalent in Australia [1]. While there have been some improvements in community understanding of common mental illnesses (particularly depression and anxiety), there is still widespread misunderstanding and ignorance [2, 3]. In particular, complex mental illnesses, such as schizophrenia, bipolar disorder and personality disorders, tend to be poorly understood and attitudes are much less positive. The low prevalence of these mental illnesses means that most people do not personally know someone with these illnesses, so they are more likely to rely on stereotypical attitudes. Common stereotypes about people with complex or severe mental illness include are that they are dangerous, unpredictable, lack competence to look after themselves, and have little chance of recovery [4]. Negative attitudes lead to discriminatory behaviour, primarily avoidance and exclusion, as people seek to avoid the risks of associating with people with mental illness. This can affect a person with mental illness’ opportunities for finding and keeping a job and their relationships with friends, family, and romantic partners [5]. This discrimination can increase feelings of worthlessness, hopelessness about the future, and suicidality [6, 7]. Reducing stigma and discrimination is therefore critical to improving the wellbeing of people with mental illness and their carers.

Reducing stigma towards people with complex mental illness is a key priority area of Australian national mental health policy. The Fifth National Mental Health and Suicide Prevention Plan (the Fifth Plan), released in 2017, focuses on stigma reduction as one of eight priorities for mental health reform [8]. It outlines the need for a nationally coordinated strategy to address stigma and discrimination and requires that the Australian government build on existing initiatives, including the evidence base of what works in relation to reducing stigma and discrimination. A recent meta-analysis of randomised controlled trials evaluated the evidence of interventions to reduce stigma towards people with severe mental illness (schizophrenia, psychosis or bipolar disorder) [9]. This found that both contact- and education-based interventions showed small-to-medium immediate reductions in stigma, but there was limited evidence on longer-term effects. There was also little guidance on what components of interventions are needed for effective stigma reduction. Furthermore, only two interventions had been evaluated in Australia, one of which was only available as part of a university experiment. While the review focused on high-quality randomised trial evidence from an international perspective, there is a need to understand what programs and initiatives are currently available in Australia specifically, and whether they have any evidence of effectiveness, even if not from randomised trials. This information is critical to inform options for a national stigma and discrimination reduction strategy as part of implementation of the Fifth Plan in Australia.

The aim of this study was therefore to (1) identify existing programs or initiatives run by Australian lived experience groups and other key non-government organisations that aim to reduce stigma and discrimination and promote positive behaviours towards people with complex mental illness; and (2) examine the evidence of effectiveness for these programs.

## Method

In order to review existing Australian stigma and discrimination reduction initiatives and their evidence of effectiveness, we conducted literature searches and surveyed lived experience groups and key non-government organisations (NGOs).

### Program inclusion/exclusion criteria

Programs were eligible if they (1) aimed to reduce stigma towards people with complex mental illness, defined as schizophrenia, psychosis, personality disorder, or bipolar disorder; (2) they focused on nonspecific ‘mental illness’ but were conducted in settings relevant to individuals with the above diagnoses (e.g., public mental health services, with mental health nurses); (3) they included the above diagnoses in program content; (4) stigma reduction was explicitly mentioned as a focus, or was implied (e.g. by including a stigma measure as an outcome or by focusing on improving understanding or knowledge of severe mental illness). All kinds of stigma were eligible, including personal or public stigma, perceived stigma, desire for social distance, discrimination, self/internalised stigma, and beliefs about recovery or prognosis.

Programs were ineligible if they (1) focused on common mental disorders (depression or anxiety), suicide, eating disorders, dementia, intellectual disability, PTSD, OCD, substance misuse or dual diagnoses; (2) aimed to improve mental health literacy or promote help-seeking without a specific focus on reducing stigma and discrimination; (3) were not conducted in Australia.

### Literature search

A systematic search of the ‘grey’ and academic literature was conducted to identify Australian programs that aim to reduce stigma and discrimination.

#### Academic databases

For the academic databases we searched PubMed and PsycINFO, limited to studies published since 2009 to ensure that they were relevant to current practice. Literature search strategies were developed using medical subject headings (MeSH) and text words related to stigma and discrimination (see Additional file 1: Table S1). All study designs were eligible including quantitative (e.g. uncontrolled trials) and qualitative (e.g. participant interviews). A total of 652 studies were screened for eligibility.

These searches were supplemented by screening our results from a previous literature review [9] to identify any reports that did not meet the inclusion criteria for that review (e.g. due to lack of a control group) but met the inclusion criteria for this review.

#### ‘Grey’ literature

The ‘grey’ literature search was conducted using Google Australia. The purpose of the ‘grey’ literature search was to identify eligible programs and to identify organisations with potential programs to be invited to participate in the survey.

Separate searches were conducted using the following key search terms: bipolar, personality disorder, (schizophrenia OR psychosis), (mental illness OR mental health), (stigma OR discrimination), and Australia. For each search, the first 50 websites were retrieved, and duplicates were excluded. The remaining websites were reviewed for relevant information and any links from these websites were followed when they were thought to contain useful information.

We also systematically searched websites of lived experience advocacy and support groups and other key NGOs to identify programs and evaluation reports. Overall, a total of 267 websites were searched for eligible programs.

### Survey of lived experience groups and key NGOs

We conducted an online survey of lived experience advocacy and support groups and key NGOs, inviting them to provide details of their programs and associated evaluation or evidence of effectiveness.

#### Survey participants

Survey participants comprised key informants in Australian organisations of any type that have programs that aim to reduce stigma and discrimination and promote positive behaviours. These were reached in 4 key ways: (1) An email sent to organisations identified in web searches (see above); (2) Information about the study with a link to the survey included in the following organisations’ newsletters: Mental Health Australia, Mental Health Victoria, and Mental Health Coordinating Council; (3) An email sent to all voting and non-voting members of Mental Health Australia. Mental Health Australia is the peak, national non-government organisation representing the interests of the Australian mental health sector. Its members include national organisations representing consumers, carers, special needs groups, clinical service providers, public and private mental health service providers, researchers and state/territory community mental health peak bodies; (4) Snowball sampling—survey respondents were encouraged to pass on details of the project to other organisations with programs that met the inclusion criteria. In total we invited 177 organisations to participate in the survey.

#### Survey content

Survey data were collected online using Qualtrics software with both multiple choice and open-ended questions. The survey included information such as location, target audience, type of program, program delivery mechanisms, program reach and source of funding. Organisations were able to provide information about multiple stigma-reduction programs, if relevant. Organisations were asked to provide any available evaluation or evidence of effectiveness. Participants provided informed consent before completing the survey. The survey opened 9th of December, 2019 and closed on 31st of January, 2020.

A concerted effort was made to obtain missing information about programs from those identified in our searches and from completed surveys. Authors of academic papers were emailed to enquire about whether programs were still operating and to obtain information not reported in the scientific literature. Organisations were also sent reminder emails to undertake or finish completing the survey before it was closed.

### Data analysis

Key relevant data from programs identified from the literature search and survey were extracted and synthesized descriptively and thematically. Level of evidence for each program was classified on a scale from 1–5, with 1 = no evaluation evidence, 2 = post survey feedback or qualitative interviews, 3 = one or more uncontrolled trials or repeated cross-sectional surveys, 4 = one or more controlled trials, 5 = one or more randomised controlled trials.

## Results

**Fig. 1:**
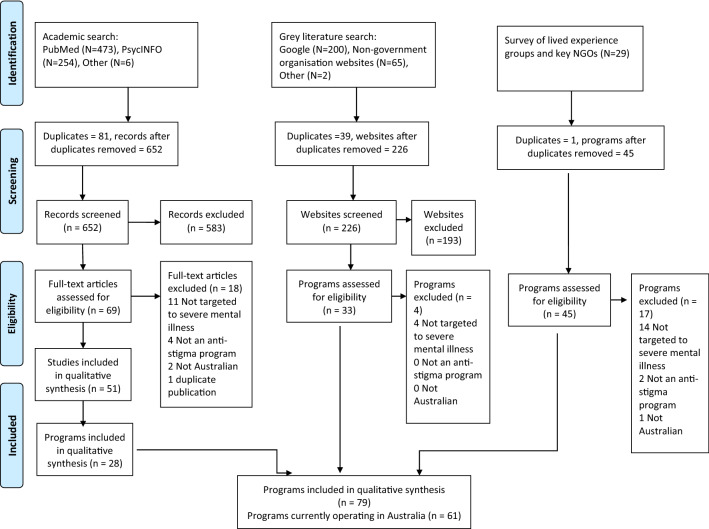
Flow chart for identifying eligible programs

Results from our survey of organisations in the mental health sector, grey literature search, and search of academic literature, identified 79 Australian programs or initiatives. These 79 programs were described or evaluated in 108 resources (as some programs were included in multiple academic papers). However, some of the identified programs did not appear to be currently available, based on information from program authors or a web search for further information. Programs that were one-offs conducted in the past, had ceased operating, or were experimental research studies not designed to be ongoing, are included in supplementary material (Tables 2 and 3). Excluding these programs left 61 programs currently operating in Australia. See Fig. 1 for a flow chart of the process of identifying eligible programs. These were further broken down into face-to-face programs (n = 29), community awareness campaigns (n = 8), programs or organisations undertaking advocacy for the rights of people with mental illness (advocacy programs, n = 5), and publicly-available online resources (n = 19).

### Face-to-face programs

Face-to-face programs were primarily targeted to four types of audiences: (1) Health professionals and health professional students; (2) People with a mental illness; (3) Family of people with a mental illness; (4) Members of the general population (particularly at school, university, or workplaces). See Tables 1, 2, 3 and 4 for characteristics of each included program.

**Table 1:** Programs targeted to health professionals, health professional students, emergency workers

Program name	Organisation	Type of mental illness	Target audience	Program description	Anti-stigma component	Lived experience involvement	Session length, facilitated by	Where provided	Duration and reach	Funding	Level of evidence^a^
Recovery Camp [14–17]	Recovery Camp	Mental illness (non-specific)	Nursing students	A non-traditional placement for nursing students. Health students and people with a lived experience of mental illness attend a recreation camp, participating in an adventure activities program in the Australian bush	Contact: Lived experience attendees are encouraged to share their stories related to mental health and recovery with students. Everyone at camp is of equal status and contact is outside an acute setting (recovery focused)	LE person was involved in program development and delivery. Previous attendees with LE are involved in designing future camps and choosing camp activities	5 days, 4 nights. Facilitated by registered nurses. Camps of 40–130 people, including 40 students, 40 people with lived experience, 5 nurse facilitators, several other staff	NSW, metropolitan	Since 2013. 800 students, 690 lived experience	Earned income from universities	4
Recovery for mental health nursing practice [18–21]	School of Nursing and Midwifery, Central Queensland University	Mental illness (non-specific)	Nursing students	A nursing subject ‘Recovery for mental health nursing practice’ introduces students to a recovery approach to mental health care	Contact: Subject is taught by an academic with lived experience	LE person was responsible for all aspects of the subject (e.g. development of content and appropriate resources, writing and examining the assessment tasks)	N/R. Subject taught by nurse with lived experience	QLD, regional/ rural	N/R	N/R	2
Remind Training and Education [22–24]	Faculty of Pharmacy, University of Sydney	Schizophrenia, depression	Pharmacy students	Pharmacy students attend a tutorial with trained mental health consumer educators, receive a series of mental health lectures and undertake supervised weekly placements in the community pharmacy setting	Contact: Consumer educators discuss their history with mental illness, the medications they take, ways of coping with their illness, the important role that pharmacists need to play in supporting people with mental illnesses, and how they were real people who led normal lives despite their illness. Students given opportunity to interview the educators during the tutorial	Trained mental health consumer educators from the Schizophrenia Fellowship of NSW participate in each session	Contact session is 2 h. Facilitated by pharmacy tutors	NSW, metropolitan	Since 2010, approx. 2,500 students	N/R	3
Collaborative Recovery Training Program (CRTP) [25, 26]	Illawarra Institute for Mental Health, University of Wollongong	Severe and persistent mental illnesses, such as schizophrenia	Health professionals	Involves training in recovery concepts and skills supporting consumers’ abilities to set, pursue and attain personal goals	Education: Aims to improve mental health workers’ attitudes towards prospect of recovery	N/R	2-day training, facilitator not reported	NSW, regional/ rural	N/R	N/R	3
Managing Mental Health Emergencies short course [27]	Australian Rural Nurses and Midwives	Range of disorders including psychosis, schizophrenia, or bipolar disorder	Rural and remote health professionals	Management of mental health emergencies including differentiating between substance intoxication and psychosis	Education: To upskill generalists in rural and remote areas to respectfully and effectively manage mental health emergency care	N/R	2-day training, facilitator not reported	Australia-wide, regional/ rural, remote	Since 2003. As of 2007, 745	Commonwealth Department of Health	3
Mental Health Intervention Team training [28, 29]	NSW Police Force, Queensland Police Service	Mental illness (non-specific)	Police officers	Training to become accredited specialist Mental Health Intervention Officers. Provides a practical skillset to assist them with managing persons within the community who are experiencing a mental health crisis event or suicidal ideation	Education: Training to identify signs and symptoms of mental illness, provide tools for communication strategies, risk assessment, de-escalation and crisis intervention techniques, and gain an understanding of the current Mental Health Act Contact: Lived experience component presented by panel of mental health consumers and a carer	N/R	4-day training (intensive), 1-day training, facilitator not reported	NSW, ACT, WA, QLD	In NSW since 2007 [4-dayprogram]. As of 2015, 2,600 officers trained. Since 2014 [1-dayprogram]. As of Dec 2015, 16,141 officers trained. In QLD since 2006	State government	4
Mental Health Intervention Team training (brief) [30]	Oak Flats VKG Call Centre	Mental illness (non-specific)	Emergency service communication officers	A brief version of the MHIT training which teaches how to respond effectively during mental health emergencies with the aim of diversion from jail to mental health treatment	Education: Training to increase the likelihood of call takers identifying mental health calls in order to prepare the responding officers before arriving at the scene	N/R	1.5–2 h, facilitator not reported	NSW, metro, regional/ rural	Since 2011, N/R	N/R	4

1 = No evaluation evidence, 2 = Post survey feedback or qualitative interviews, 3 = One or more uncontrolled trials or repeated cross-sectional surveys, 4 = One or more controlled trials, 5 = One or more randomised controlled trials

*LE* Lived Experience, *N/R* Not Reported

**Table 2:** Programs targeted to people with mental illness

Program name	Organisation	Type of mental illness	Description	Anti-stigma component	Lived experience involvement	Number of program attendees	Where provided	Duration and reach	Funding	Level of evidence
The Station [31]	The Station	Mental illness (non-specific)	Consumer-driven mental health service provides a safe and supportive environment, social connections, and activities for its members (those with a lived experience of mental illness). Aims to increase knowledge and skills for living	Contact: People recovering from a mental illness, their carers, and community members meet and conduct activities. Targets public stigma and self-stigma (self-worth)	People with LE involved in all aspects of service delivery and are part of the management committee	50 people	SA, regional/ rural	Since 1998, N/R	State gov, earned income from members, donations	2
TasRec	Richmond Fellowship Tasmania	Mental illness (non-specific)	Recreation program provides a broad range of creative, social and skills building activities to help support mental wellbeing, build confidence and self-esteem, reduce isolation	Contact: The recreation program uses community events and art shows to convey experiences of mental illness and their capacity to lead meaningful lives whilst living with illness. Consumers are also provided the opportunity to increase their community engagement through participation in a wide variety of recreation activities, including physical, health, art, and so on. Targets public stigma and self-stigma (self-worth)	Recreation program is a process of co-design and collaboration between people with LE and staff within the programs. LE provide suggestions for activities and tasks they would like to participate in	Depends, small groups generally	TAS, metro, regional/ rural	5–10 years, 140 people	Commonwealth gov	1
Residential Accommodation	Richmond Fellowship Tasmania	Mental illness (non-specific), Bipolar disorder, Personality disorders, Psychosis, Schizophrenia	Residential accommodation for consumers living with mental health issues. RFT provide supports to consumers enabling them to reach greater independence, combat stigma, increase their personal advocacy, and live meaningful lives	Other: Consumers are encouraged to envision the lives they wish to lead, and are provided examples of others leading meaningful lives, in the presence of mental illness. They are supported to access services, build social networks and lead meaningful lives despite stigma associated with mental ill-health Protest/Advocacy: Consumers are supported to build resilience and learn to advocate for themselves, as individuals navigating complex systems and situations	People with LE participate in consumer advisory council and co-design and collaboration of service building	25 people	TAS, metro, regional/rural, remote	More than 10 years, hundreds of participants	State gov, earned income from residents	2
Compeer (The Friendship Program) [32]	St Vincent de Paul Society Canberra	Mental illness (severe)	Friendship between a volunteer and person with lived experience who are matched based on age, gender, interests, hobbies and availability	Contact: Matches meet weekly for one year in safe environments using natural supports, sharing decision-making around activities, place, and time	Volunteer members of the public meet people with LE to develop friendships	20–25 participants in 2020	NSW, ACT, metro, regional/rural	Since 2009, 253 participants (ACT branch)	State gov (ACT)	2
Hearing Voices group [33]	Uniting Prahran	Schizophrenia	Monthly/fortnightly peer support group provides a welcoming space for voice hearers to share what it’s like to hear voices, learn new coping strategies and explore ways to make sense of voices and to change the relationship with voices	Other: The focus of the group is on support. Individuals are provided with the chance to share their experience of hearing voices and ideas of living with the voices	Facilitators are a person with LE and a ‘worker’	N/R	VIC, metro	N/R	N/R	1
Information Nights	Borderline Personality Disorder Community	Borderline Personality Disorder	Information Nights are held three times a year to the BPD Community to provide information, a forum for discussion, and a sense of community	Contact: Some information nights feature people with LE sharing their stories to reinforce the core techniques that build relationships and recovery Education: Information nights aim to replace stigma and discrimination with the hope and optimism that recovery is a realistic goal. Speakers present on topics of interest to the BPD Community Protest/Advocacy: Aim to increase capacity for advocacy through information and relationships with individuals in the community	Facilitators are a person with LE, carer	Average of 28 over the last 5 events	VIC, metro	Since 2014, at least 167 people	Volunteer	1
My Recovery	Northern Territory Mental Health Coalition	Mental illness (non-specific)	A peer-led recovery program delivered by peers to other people with experiences of mental health challenges	Contact: Peer led Education: Sessions cover information on mental illness, stigma and discrimination, recovery and discrimination, as well as skills-based capacity building in communication, recovery, and goal setting to promote long-term mental health and wellbeing Protest/Advocacy: Types of advocacy and local advocacy services are covered in sessions	Facilitators are a person with LE	12 to 15 people	NT, metro	6–12 months, 30 people	Commonwealth gov	2
Being Herd [34]	Batyr	Mental illness (non-specific)	A workshop where young people are trained to share their stories to help breakdown the stigma associated with mental health	Other: 2-day workshop aims to enable people with lived experiences to tell their story in a constructive and empowering way. Highlights steps the person took to get support, what has helped in their recovery and how they can share their story in a safe and effective way for themselves and other young people	Facilitators are not reported	N/R	N/R	700 + young people	N/R	1

1 = No evaluation evidence, 2 = Post survey feedback or qualitative interviews, 3 = One or more uncontrolled trials or repeated cross-sectional surveys, 4 = One or more controlled trials, 5 = One or more randomised controlled trials

*LE* Lived Experience, *N/R* Not Reported

**Table 3:** Programs targeted to family of people with mental illness

Program name	Organisation	Type of mental illness	Description	Anti-stigma component	Lived experience involvement	Session length, facilitated by	Where provided	Duration and reach	Funding	Level of evidence
Family and friends group	BPD Community	Borderline Personality Disorder	A group for carers to provide support and psychoeducation. Groups aim to share and learn how to support each other; to actively seek education and training to improve our relationships with our loved ones; to help ourselves and others; to create a safe environment; to reduce our sense of isolation; to accept our individual and joint responsibility to this purpose	Contact: Groups are spent sharing stories over the month Education: One hour of the meeting is devoted to learning about relevant topics Protest/advocacy: The group provides the opportunity for individuals to build their own advocacy. It also provides the organisation with the capacity to speak on the behalf of participants	Program is designed and developed by carers with LE	2.5 h once a month, facilitated two carers	VIC, metro	Since 2015, 167	Volunteers	Unclear
Journey to Recovery [35–38]	St Vincent’s Mental Health Service	Psychosis	Psychoeducation group program in a public adult mental health service for the families and friends of people experiencing early psychosis	Education: Provide support and information to assist coping and reduce isolation. Topics include What is psychosis, Recovering from psychosis, Medications, Early warning signs (relapse prevention), Community resources	None reported	5 × 2-h sessions. Inpatient version is a single session. Facilitated by early psychosis senior clinicians	VIC, metro	Since 2009, N/R	State gov	3
Kookaburra Kids Camps and Activity Days	Australian Kookaburra Kids Foundation	Mental illness (non-specific)	Therapeutic recreation camps and activities for children who are living with a family member affected by mental illness	Education: Psychoeducation and basic coping skill-building is embedded into programs in a supported peer-group format to promote mental health literacy (including addressing misconceptions and myths about mental illness) and appropriate help-seeking	Designed by person with LE, co-design committee initiated in 2019. Delivery includes volunteers with LE	2 × 1-h groups at camps; 15 min psycho-ed and activity at Activity Day. Facilitated by trained staff	ACT, NSW, NT, QLD, SA, VIC. Metro, regional/rural	More than 10 years, 3,000 +	Govt, donations and corporate / other sponsorships	Unclear

1 = No evaluation evidence, 2 = Post survey feedback or qualitative interviews, 3 = One or more uncontrolled trials or repeated cross-sectional surveys, 4 = One or more controlled trials, 5 = One or more randomised controlled trials

*LE*Lived Experience, *N/R* Not Reported

**Table 4:** Programs targeted to the general population

Program name	Organisation	Type of mental illness	Target audience	Program description	Anti-stigma component	Lived experience involvement	Session length, facilitated by	Where provided	Duration and reach	Funding	Level of evidencea
Mental Health 101 (Youth/Adult) [39–42]	Mental Illness Education ACT (MIEACT)	Mental illness (non-specific)	Youth program targets high school students (years 7–10). Adult program targets workplaces	Workshop providing an introduction to mental health. Stigma-based learning outcomes include an understanding of what stigma is, being able to identify negative consequences of stigma, and an ability to contribute to the collective impact to reduce stigma in relation to mental illness	Contact: Two volunteer educators with lived experience share stories of living with a mental illness Education: an understanding of myths and facts about mental health and examples of help-seeking behaviours	Programs are delivered by people with LE. Programs are co-designed with mental health professionals and people with LE	1 60-min session, facilitated by person with lived experience	ACT, metro, regional/rural	Since 1993, 8,000 people per year	Commonwealth gov, state gov, and private funding	4
Mental Health First Aid [43–53]	Mental Health First Aid Australia	Mental illness (non-specific), Bipolar disorder, Psychosis, Schizophrenia, Depression, Anxiety, Substance Misuse, Non Suicidal Self Injury	General population	A program which teaches members of the public how to provide mental health first aid to others and enhances mental health literacy. A variety of courses exist: Standard MHFA (for adults), Youth MHFA (for adults assisting young people), Older Person MHFA, Aboriginal and Torres Strait Islander MHFA	Contact: Two videos involve people with lived experience of mental illness talking about their experiences (one psychosis, one anxiety). Majority of instructors share their own experiences in their teaching Education: Provides accurate information about mental illness to bust myths (e.g. that people with psychotic illnesses are dangerous and unpredictable) Hallucination simulation: Optional activity where two volunteers have a discussion whilst the instructor reads from a scripted ‘voice’	Founder has lived experience of mental illness. Curriculum based on consensus studies involving people with lived experience (consumers and carers). Courses are delivered by instructors, most of whom have lived experience as consumers or carers	Standard MHFA is 12 h, Youth MHFA is 14 h. Training is facilitated by an instructor who is accredited by MHFA Australia. Instructors	Australia-wide, metro, regional/rural, remote	Since 2000, 800,000 people	Varies according to Instructor. MHFA Australia receives earned income, intermittent funding from government and philanthropic sources	5
Peer Ambassador Program	SANE Australia	Mental illness (non-specific), Bipolar disorder, Personality disorders, Psychosis, Schizophrenia, Eating Disorders, Suicide, other low prevalence disorders including complex trauma	General population	SANE Peer Ambassadors are a group of people who work with SANE Australia to raise awareness, reduce stigma and provide hope to Australians affected by complex mental illness. They also help develop, deliver and evaluate SANE’s programs and services. All Peer Ambassadors receive training and support, guiding them through the process of sharing their story in ways that align with their reason for becoming an ambassador	Contact: Presentations in workplaces and community settings to share their personal experience of living with, or supporting someone with a complex mental illness. Online stories via SANE website Protest/Advocacy: Participants are regularly invited to contribute to advocacy and research projects, review resources and provide their insights through co-design or research projects	People with LE are paid staff on the program. Program was relaunched in 2018 following extensive consultation with people with LE	1 45-min session, facilitated by person with LE	Australia-wide, metro, regional/rural, remote	Since 1986 in various forms, 1,000 + (currently 110 Peer Ambassadors)	Corporate partnerships	1
Batyr (@school, @uni, @work) [54, 55]	Batyr	Mental illness (non-specific)	High schools, universities, workplaces	Programs delivered to schools (batyr@school), universities (batyr@uni), and workplaces (batyr@work)	Contact: Two people with lived experience share their stories, focusing on help-seeking journey [10 mineach]. Video stories are in development and only used in rural communities Education: Signs of mental illness, how to support a peer, seek help, role of language in perpetrating stigmatising attitudes Protest/Advocacy: An addition to the School program, school chapters empower 20 passionate students to lead mental health events on their own school campus throughout the year	Lived Experience speakers form part of the governance of batyr, and are instrumental in any decision made within the organisation	1 session 60–90 min, facilitated by person with lived experience and other trained person	ACT,NSW,QLD,SA,VIC, metro, regional/rural, remote	5–10 years, 229,934 people	Earned income	5
SPEAK UP! Stay ChaTY [56, 57]	SPEAK UP! Stay ChaTY	Mental illness (non-specific)	High schools, sports/arts organisations, workplaces	Education and awareness programs. Stay ChatTY Schools Program to grades 9–12, Stay ChatTY Sports Program to sporting clubs, Community Presentation to workplaces and community groups	Contact: Founder Mitch McPherson shares his personal story of losing his brother to suicide through his lived experience story. Lived experience videos of community members sharing their stories of mental ill-health and suicide are used in the Sports Program, the Schools Program and online Education: Programs teach information on mental health vs mental illness, stigma, signs and symptoms of mental illness, resilience, where to access support, helping a friend/team mate/ Other: Delivers online anti-stigma and awareness campaigns via social media, engages with community partners for wellbeing and awareness events, attends community expos and events to promote anti-stigma messages	Founder with LE supports program development. A Youth Reference Group includes a number of young people with lived experience informs the development of youth-focused program content	1 45–90 min session. Facilitated by person with lived experience, nurse, exercise physiologist, lawyer, researcher	Australia-wide, metro, regional/rural, remote	Since 2013, ~ 25,000	State gov, donations, community grants	3
LIVINWell [58]	LIVIN	Mental illness (non-specific)	Organisations (e.g. workplaces, universities, schools, sports/arts organisations)	Introductory mental health awareness program to educate people on a range of issues related to mental health, with an emphasis on breaking the stigma of mental health, enhancing self-efficacy and encouraging help-seeking behaviour	Contact: In-person stories of facilitators’ lived experience with mental illness. Video stories of co-founders and how/why LIVIN originated and what their mission is Education: Accurate alarming statistics on mental illness and suicide in Australia	Programs are co-delivered by people with LE	1 45-min session, facilitated by mental health professionals and person with lived experience	Australia-wide, metro, regional/rural, remote	5–10 years, N/R	N/R	1
Mental Health Awareness	Mental Health Partners	Mental illness (non-specific)	Organisations (e.g. workplaces, universities, sports/arts organisations)	Short courses delivered to private organisations to reduce stigma, give information, offer resources and improve mental health	Contact: Courses include at least one person with LE who shares their story to inform participants. Most courses include video of people with LE explaining their journeys Education: Myths and facts sessions to improve knowledge	Programs are designed and co-delivered by people with LE	1 3-h session, facilitated by social worker, person with lived experience	Australia-wide, metro, regional/rural, remote	2–5 years old, 1,200 participants	Earned income from private organisations	2
Staff Wellbeing Workshop [59, 60]	Chess Connect	Mental illness (non-specific)	Workplaces	A workshop that helps employers collaborate with their staff to educate and promote a positive mental wellness workplace culture	Education: Program covers understanding stress, active stress management, reducing stigma, understanding the link between life events, the brain and behaviour, building resilience practices, understanding the impact of workplace habits, and recognising when a person is unwell or struggling	N/R	1 2-h session, facilitated by ‘Workplace Wellness specialist’	NSW, regional/rural	N/R, Over 750 people	N/R	1
Exhibition Program [61, 62]	The Dax Centre	Mental illness (non-specific)	General population	Exhibition Program of art by people with lived experience open to the general public	Education: The exhibition may include bios written by the artists which allow the artist to share aspects of their lived experience that break down myths and provide accurate information about mental illness for visitors	All artists that exhibit have a lived experience and are involved in the process of exhibition development	People visit for between 10 and 20 min. Guided tours last between 30 and 60 min. Facilitated by staff at the Dax Centre	VIC, metro	More than 10 years, ~ 24,000	Commonwealth gov, philanthropic, earned income	2
Education Program (Mindfields) [61, 63]	The Dax Centre	Mental illness (non-specific)	Universities, schools	A range of education programs specifically tailored to secondary and tertiary students who are studying mental health or arts-related subjects, encompassing presentations from advocates with LE and tour of current exhibitions	Contact: Advocates present to the students sharing their lived experience of mental health issues, including a discussion of symptoms, their journey relating to diagnosis, treatment and other recovery factors. Some programs include video stories. Exhibition tours also include information on the artists’ personal stories Education: Myth-busting is woven into the guided tour of exhibitions. Information is given about the history of psychiatric care in Victoria and how stigma has impacted community understanding over time	Programs delivered by people with LE. Advocates provide feedback on the program and how it can be designed to be more effective	1 2-h session, facilitated by people with LE, neuroscientists	VIC, metro	More than 10 years, 22,000 people	Commonwealth gov, philanthropic, earned income	1
Mental health awareness forums	Australian Rotary Health	Mental illness (non-specific), Bipolar disorder, Personality disorders, Psychosis, Schizophrenia	General population	Community forums, organised by Australian Rotary Health and Rotary Clubs, to discuss all aspects of mental health. Speakers usually a mental health professional, a consumer and a carer. Members of the general public are invited to attend	Contact: Members of the community who have a mental illness are invited to attend and speak Protest/Advocacy: Holding a public forum provides advocacy for mental health awareness and acceptance. No specific activity is undertaken except openness and general discussion on mental health	People with LE are invited to speak when the program is arranged	1 2-h session, facilitated by various people, e.g. health professional, Rotarian, MP	Australia-wide, metro, regional/rural, remote	Since 2000, ~ 5000 people	Commonwealth gov (now ceased), some private	2

1 = No evaluation evidence, 2 = Post survey feedback or qualitative interviews, 3 = One or more uncontrolled trials or repeated cross-sectional surveys, 4 = One or more controlled trials, 5 = One or more randomised controlled trials

*LE* Lived Experience, *N/R* Not Reported

About half (55%) of the face-to-face programs focused on stigma towards people with a non-specific mental illness, six (21%) targeted a range of disorders including psychosis, schizophrenia, bipolar disorder or personality disorders, three (10%) specifically focused on psychosis or schizophrenia, two (7%) on ‘severe’ mental illness, and two (7%) specifically on Borderline Personality Disorder.

Three-quarters (76%) of organisations providing anti-stigma programs were classified as not-for-profit or community sector, and the remainder were government (10%), university/tertiary education (10%), or private/for-profit (3%). A majority of organisations (66%) provided a range of services, including some anti-stigma programs, rather than only running anti-stigma programs (34%), and a majority reported running multiple anti-stigma programs (62%). A minority of programs were run in all Australian states and territories (24%), with the largest number run in NSW (31%), followed by Victoria (28%), the ACT (17%), Queensland (14%), South Australia (10%), Tasmania (10%), Northern Territory (10%) and Western Australia (3%)). Programs were also delivered across metropolitan (72%), regional and/or rural areas (62%) and remote communities (31%) with half delivered across multiple geographic areas.

Programs were delivered in a variety of settings, most commonly community settings (e.g. sports or arts organisations, 45%), followed by community health centres (41%). Also common were workplaces (38%), university or tertiary education settings (34%), primary healthcare (17%), and high school (14%). Only 2 were run in primary schools (7%). Programs tended to target adults (59%) or ‘all ages’ (14%). Adolescents were the target age group in four programs (14%) and young adults in two (7%). In addition, one program targeted children 8–18 years old (3%).

Most programs involved people with lived experience in their design (59%) or delivery (76%). Programs often included multiple types of components, but the most common was an education component (66%) followed by face to face contact (62%) or online/video contact (24%). Protest or advocacy was reported in 24% of programs. Only one program included an (optional) hallucination simulation component (3%).

Seven programs did not report a funding mechanism. Of the remainder, there was a variety of funding sources. Funding was sourced most frequently from the Commonwealth government (25% of reported) or from earned income (22%), followed by state government (19%), donations or volunteers (11%), philanthropic (8%), corporate sponsorship (6%), and other means (8%).

Most of the programs were well-established, with half running for more than 10 years (48%), 28% running for 5–10 years, one was 2–5 years old (3%), and one was 6–12 months old (3%). This information was not reported or available for nearly a fifth of programs, however. Information about program reach was not available for seven programs. Of the remainder, ten (45%) had reached up to 1000 people, five (23%) 1000–10,000, four (18%) reached 10,000–100,000, and three programs (14%) had reached over 100,000 people.

The level of evidence for most programs was low. Seven programs (24%) reported no evaluation evidence and a further eight (28%) were evaluated with post program surveys or qualitative interviews only. These surveys tended to focus on satisfaction outcomes rather than impact on stigma. Only two programs (7%) were evaluated with one or more randomised controlled trials, the highest level of evidence. Six programs (21%) had one or more controlled trials, four (14%) were evaluated with one or more uncontrolled trials or repeated cross-sectional surveys, and for two programs the type of evaluation was unclear. Information about program evaluations is available in Table 5.

**Table 5:** Evaluation data from face-to-face programs

Program name	Experimental design	Study sample	Sample size	Measures	Outcomes
Batyr [55]	RCT	N/R	N/R	N/R	In 2017, Macquarie University conducted a study into the effectiveness of the batyr@school program, looking at stigma reduction and help-seeking. The biggest two findings were 1. The program was successful in reducing stigma that young people had towards others experiencing mental health issues 2. The program lead to an increase in attitudes and intentions towards seeking help from professional sources for mental health issues and suicidal thoughts The findings were maintained for at least 3 months after the program
BPD Community Information Nights	Post feedback	N/R	N/R	N/R	Usefulness of the event and information: 99% find them useful Personal confidence and understanding: 83% said its better Feeling more supported: 80% said yes Help personal ability to build relationships: 92% yes Do you expect to use knowledge gained: 97% said yes
BPD Community Family & Friends Group	N/R	N/R	N/R	N/R	From program authors: “A ‘formal’ evaluation occurred in 2017 which lead to the evolution of the program of today. Monthly evaluations of the program are conducted”
Collaborative Recovery Training Program (CRTP) [25]	Uncontrolled trial (pre/post)	Mental health workers from government and NGO organisations in eastern Australia	75 with data to analyse out of 103	Staff Attitudes to Recovery Scale (STARS; Crowe et al., 2006) assesses hopeful attitudes regarding consumers’ recovery possibilities. Therapeutic Optimism Scale assesses treatment expectancies	There was an improvement in STARS pre-post (d = 0.87) and therapeutic optimism scores pre-post (d = 0.78). MANOVA p = .02
Compeer (The Friendship Program) [32]	Survey only	Volunteers from the Compeer program	72 analysed	Social Distance Scale, Affect Scale, Dangerousness Scale, Match Bond (measures friendship strength)	A stronger relationship between the Compeer volunteer and friend was associated with lower levels of stigma: social distance (p = .001), Affect (p = .015), Dangerousness (p = .028). No relationship between time spent in relationship and stigma, suggesting it is quality of contact rather than length of contact that reduces stigma
Journey to Recovery [37]	Uncontrolled trial (pre/post)	Carers of person with psychosis	15	6 questions on perceived knowledge: understanding of psychosis, understanding of recovery, knowledge of medication, relapse prevention, understanding of links between substance use and psychosis, plus qualitative feedback	Significant improvements in perceived knowledge of psychosis (p = .001) and recovery (p = .008) pre to post. Qualitative feedback was that participants valued support, felt a reduced sense of isolation, felt a sense of collective experience, and appreciated the opportunity to ventilate and feel heard by peers
Journey to Recovery [35]	Qualitative interviews	(1) carers who continually attended; (2) carers who attended once only; (3) carers who never attended; (4) case managers and (5) early psychosis clinicians	10 carers, 8 clinicians	7 qualitative questions designed to illicit positive and critical information and suggestions for the future direction of the group	Carers reported Reduced isolation, sense of Collective Experience, Opportunity to vent and feel heard, Reduced stigma and shame, Increased knowledge about mental illness, Enhanced skills in supporting the person experiencing mental illness. The group enabled “helping us to communicate as a family again,” “learning how to communicate and describe what mental illness is to our children,” and “passing it on into the community to help others” (reduced stigma and shame)
Journey to Recovery (inpatient version) [36]	Qualitative interviews 6 months later	Carers of person with psychosis	27	14-item interview questionnaire on timeliness, correct people invited, sufficient time, useful information (written, oral, DVD, booklet, fact sheets), support offered, family use of information, follow-up in community, and improvement suggestions	The session and materials were perceived as helpful. Findings in the present study suggest that early psychosis carers are open to receiving psychoeducation at first contact with psychiatric services
Journey to Recovery [38]	Uncontrolled trial (pre/post)	Families of people with early psychosis	17	6 questions on perceived knowledge: understanding of psychosis, understanding of recovery, knowledge of medication, relapse prevention, understanding of links between substance use and psychosis, plus qualitative feedback	Significant improvements in perceived knowledge of psychosis and recovery pre to post (ps < .001). Qualitative feedback was that participants valued peer support and support from session facilitators, felt a reduction in a sense of isolation, felt a sense of collective or similar experiences and there was an appreciation of the opportunity to ventilate feelings and be heard by peers who understood the challenges faced
Kookaburra Kids camps and Activity Days	N/R	N/R	N/R	N/R	From program authors: “Evidence of impact; (changes in MHL and help seeking) currently continuing with published research to follow 2020”
Managing Mental Health Emergencies short course [27]	Repeated cross-sectional surveys (pre/post with some follow-up interviews 3-6mth)	Rural and remote healthcare providers (nurses, Aboriginal health workers, other allied health)	N = 456 at pre, N = 163 post workshop, N = 44 interviews	Survey: 7 questions ranking perceived skills. No information about interview guide	Perceived skills improved in differentiating between psychosis and substance intoxication (p < .001), assessing psychotic symptoms (p < .001), communicating effectively with people with mental health problem (p < .001), assessing suicide risk (p < .001). Almost all interview participants felt they had changed their attitude towards mental health clients as a result of the course, as many recognised that had been stereotyping and stigmatising clients. Participants talked about their increased patience when listening to acutely unwell clients
Mental Health 101 [42]	Controlled trial (pre/post). Comparison condition was non-participating schools	High school students	457	Two vignettes on stigma which were followed by four questions about their attitudes towards the person described in the vignette and four social distance questions. Multiple-choice questions and open-ended questions on knowledge of mental health and mental illness, and the General Intentions to Seek Help Questionnaire	The intervention group had lower mean stigma scores (p = .000) and greater knowledge on each of the knowledge questions (all p < .001), and increased help-seeking intentions (p = .000) compared to the control group at post-test. Further analysis revealed a significant effect of the intervention on reducing stigma after the effect of knowledge was removed (p < .001) Qualitative responses revealed many students were deeply touched by the personal stories of presenters, that they were a powerful medium, and made the impact of mental illness tangible and encouraged the realisation that people with mental illness were just ‘ordinary people with extraordinary stories’
Mental Health 101 [41]	Qualitative interviews	Volunteer consumer educators	10	Semi-structured interview focused on the benefits and costs related to being in an advocacy/educator role and its impact on recovery from the experience of mental illness and treatment	Reports on the benefits and costs of being a lived experience educator in the MIE-ACT program. Benefits identified were the value of peer support where educators felt a unique sense of acceptance and understanding from their peers, gaining a sense of purpose and personal meaning from the personal satisfaction of educating others, and the impact and therapeutic effect broadcasting had in reducing self-stigma and assisting in positive identify development. Costs reported were feeling ‘raw’ or vulnerable during or after presenting and a fear of being stigmatised as a result of presenting
Mental Health 101 [42]	Post surveys	High school students (93.3%)	N/R, 90.7% of learners are surveyed after the program	Satisfaction ratings, perceived knowledge	89.7% of learners rated the program as either extremely of significantly informative 97.2% of learners state that the programs had increased their understanding of mental health
Mental Health Awareness	Post course evaluations of all programs	N/R	N/R	N/R	N/R
Mental Health First Aid [43]	RCT. Comparison condition was waitlist	Nursing students	181 (int = 92, control = 89)	Social Distance Scale, Personal Stigma Scale, Perceived Stigma Scale (all for depression vignette)	Outcomes are not relevant as not for schizophrenia/psychosis/bipolar disorder/personality disorder
Mental Health First Aid [44]	RCT. Comparison condition was waitlist	Adult members of community	178 (int = 90, con = 88)	Social Distance Scale, Personal Stigma Scale (depression and schizophrenia)	For schizophrenia, improvements pre-post in personal stigma (p < .001) and social distance (p < .001). Sig improvements at 6-mth FU: personal stigma (p < .001) and social distance (p < .01)
Mental Health First Aid [45]	RCT. Comparison condition was waitlist	High school teachers	423 (int = 283, con = 140)	Personal Stigma Scale for depression only	Outcomes are not relevant as not for schizophrenia/psychosis/bipolar disorder/personality disorder
Mental Health First Aid [46]	Uncontrolled trial (pre/post/6mth FU)	Adult members of community	246	Personal Stigma Scale and Perceived Stigma Scale (for depression and schizophrenia)	Improvements in beliefs about dangerousness (p = .005), unpredictability (p < .001), and willingness to disclose (p = .005) pre to post for schizophrenia. Changes in stigmatising attitudes about schizophrenia from pre-test to follow-up were only significant for disagreement about dangerousness (from 33.1% to 48.5%, p = 0.008). No significant change in perceived stigma
Mental Health First Aid [47]	Uncontrolled trial (pre/post)	Members of the Chinese community in Melbourne	108 (84 analysed)	Social Distance Scale (towards depression and schizophrenia vignettes)	Social distance for schizophrenia sig improved pre-post (p = .005)
Mental Health First Aid [48]	Uncontrolled trial (pre/post)	Members of the Vietnamese community in Melbourne	114	Personal Stigma Scale and Perceived Stigma Scale (for depression and schizophrenia)	Significant improvement in some personal stigma items for early schizophrenia (4 of 9) and chronic schizophrenia (3 of 9)
Mental Health First Aid [49]	Uncontrolled trial (pre/post/6mth FU)	Workers and volunteers of organisations working in multicultural communities	458	Social Distance Scale, Personal Stigma Scale, Perceived Stigma Scale (towards depression and schizophrenia vignettes)	Pre-post sig improvements in social distance (p < .001), personal stigma (p < .001) and perceived stigma (p < .001) for schizophrenia. Stigma data not collected at follow-up
Mental Health First Aid [50]	RCT. Comparison condition was Red Cross First Aid training	Australian parents of teenagers	384 (int = 201, con = 183)	Social Distance Scale, Personal Stigma Scale (Weak not sick, Dangerous/unpredictable) towards psychosis vignette	No significant changes in stigma outcomes in parents at 1-year and 2-year follow-up
Mental Health First Aid [53]	Controlled trial	Pharmacy students	272 (int = 60, con = 212)	Social Distance Scale for schizophrenia	Reduced social distance over time compared to control, p < .001
Mental Health First Aid [51]	RCT	Public servants	608 (int elearning = 199, int blended = 199, con = 210)	Social Distance Scale and Personal Stigma Scale (both for depression and PTSD)	Outcomes are not relevant as not for schizophrenia/psychosis/bipolar disorder/personality disorder
Mental Health First Aid [52]	Controlled trial (pre/post/3mthFU)	Chinese international students studying in Melbourne	202 (int = 102, con = 100)	Personal Attributes Scale, Social Distance Scale (both for depression and schizophrenia)	Significant improvements over time for social distance towards schizophrenia (p = .021). No sig change in perceived dangerousness or perceived dependency
Mental Health First Aid	Qualitative focus groups	Mental health first aid instructors, and members of the Aboriginal and Torres Strait Islander community	N/R	N/R	N/R
Mental Health Intervention Team (MHIT) training [28]	Controlled trial (pre/post/18 month FU). Comparison condition was officers who were not trained	NSW police officers, NSW health staff	260 (trained = 186, not trained = 74). Presurvey = 112, post = 32, FU = 42)	Levels of confidence, self-reported behaviour change,	The MHIT training led to an increase in confidence in dealing with jobs involving individuals with a mental health problem, or a drug induced psychosis at post and follow-up (ps < .001). Qualitative data supports the notion that the MHIT training led to an increase use of de-escalation techniques, with officers reporting that an increased understanding of mental health meant they were better able to deal with the situation. Qualitative data from NSW Health staff working specifically in mental health were uniform in their perception of an improved understanding about mental health amongst the police officers they engaged with when a scheduled consumer was delivered to their care, and noted the flow-on effect that officers ‘ increased understanding of mental health had on their engagement with consumers
Mental Health Intervention Team (MHIT) training (brief version) [30]	Controlled trial (post only). Comparison condition was those who have not completed the training	Emergency call operators (communications officers)	91 (trained = 18, not trained = 73)	Community Attitudes Towards Mental Illness (CAMI); Social Distance Scale	Findings showed no difference in stigma between those who had undergone CIT training and those who had not
My Recovery	Qualitative interviews	Lived experience adult members of the community	30 (Presurvey = 14, post = 16)	N/R	N/R
Recovery Camp [17]	Controlled trial (pre/post). Comparison condition was traditional nursing placements (inpatient and community mental health)	3rd year nursing students	50 (Recovery Camp = 23, comparison = 27)	Preplacement Survey, includes items on Negative stereotypes and Anxiety surrounding mental illness	Sig greater reduction in anxiety (p = .001) and negative stereotyping (.015) in intervention group compared to control. In particular, decreased endorsement of statements that describe mental illness sufferers as unpredictable, incapable and dangerous in the Recovery Camp group
Recovery Camp [15]	Controlled trial (pre/post). Comparison condition was traditional nursing placements (inpatient and community mental health)	3rd year nursing students	79 (Recovery Camp = 40, comparison = 39)	Social Distance Scale	Sig reductions in social distance in the Recovery Camp group pre to post, and pre to follow-up. No sig reduction in social distance in comparison group
Recovery Camp [16]	Qualitative analysis of written reflections	3rd year nursing students	20	4 critical reflections during their time at Recovery Camp	Students reported the placement was a unique, positive and educational mental health nursing placement. It allowed for the application of knowledge, consolidation of skills, experience of recovery-orientated care, development of therapeutic relationships and learning from people with a lived experience of mental illness about mental illness and related treatments. Recovery Camp was transformative in terms of learning the strengths of people with a lived experience of mental illness, acknowledging previously held fears and anxieties, and establishing future plans for practice
Recovery Camp [14]	Qualitative analysis of written reflections	3rd year nursing students	56 (28 students, 27 LE)	Content analysis of student reflective quotes	Reflective quotes of students’ experiences showed their understanding and empathy towards people with a mental illness increased, they developed practical skills, appreciated and learnt how to establish and maintain therapeutic relationships, and discovered the importance of lived experience
Recovery for mental health nursing practice [18]	Qualitative interviews	Nursing students	12	Asked to describe their views and experiences being taught by a person with LE, positives, negatives, and how their nursing practice would be influenced	Students were positive and reported an enhanced self-awareness and greater understanding of the person behind the diagnostic label and their experience. It encouraged them to question their attitudes and prejudices
Recovery for mental health nursing practice [19]	Controlled trial (pre/post). Comparison condition was traditional mental health nursing subject taught by nurse academic	Nursing students	171 (intervention = 110, comparison = 61)	Mental Health Consumer Participation Questionnaire	Both courses improved some aspects of attitudes towards consumer participation in mental health care
Recovery for mental health nursing practice [21]	Controlled trial (pre/post). Comparison condition was traditional mental health nursing subject taught by nurse academic	Nursing students	201 (intervention = 131, comparison = 70)	Scale measuring Anxiety surrounding mental illness and Negative stereotypes	The lived experience-led course showed sig decrease in negative stereotypes (p < .001). Reduction in anxiety was not sig (p = .04—p = .01 set as significance level). Reductions in comparison group were not significant (p = .02 for anxiety and p = .06 for stereotypes)
Recovery for mental health nursing practice [20]	Qualitative interviews	Lived experience educators	12	Not clear	Reports on the experience of being a lived experience educator in nursing programs. Themes identified were facing fear, demystifying mental illness and issues of power
Remind Training and Education [23]	Uncontrolled trial (pre/post/12 mth FU)	Pharmacy students	178	Questionnaire with 8 items on stigma towards schizophrenia, reported as individual items. Also focus groups with 11 participants	Significant decreases in stigma at 6-week post and follow-up for 5 out of 8 items relating to schizophrenia (p < .05) (unpredictable; have different feelings; are difficult to talk to; should pull themselves together; are not a danger to others; have themselves to blame). Focus groups showed that the intervention made mental illness more real to them and increased insight, enabled them to see consumers are able to lead a normal life despite their illness, removed some pre-conceived ideas they had about consumers, realised that pharmacists need to be non-judgemental in their interactions with consumers
Remind Training and Education [24]	Separate focus groups with students and consumers	Pharmacy students and consumer educators	23 (11 students, 12 consumer educators)	Impact of the training on students and goals, challenges and benefits of mental health consumer educators providing education to health professional students	All consumers nominated reducing stigma as a primary reason for becoming an educator. The contact the students had with the MHCE provided them with a greater insight into what it is like to suffer from psychotic symptoms and the challenges people face in managing their mental illness. Students reported a change in how they interacted with patients (pharmacy practice) and that their confidence had improved. Consumer educators felt empowered by their participation, reported improved confidence and public speaking skills, and enjoyed the social contact with other consumers. Some reported that fear of social situations was a challenge to fulfil their role
Remind Training and Education [22]	Controlled trial (pre/post). Comparison condition was film-based contact	Pharmacy students	244 (direct contact = 122, indirect contact = 122) were analysed	Social Distance Scale for mental illness [7items]; Attribution Questionnaire [6items]; 8 items on specific stigmatising beliefs towards schizophrenia	Both interventions showed similar reductions in Social Distance scores. The training had greater effect for 5 of 6 Attribution Questionnaire items and 5 of 8 stigma items. Both interventions showed reductions in stigma though
Richmond Fellowship Residential Accommodation	N/R	N/R	N/R	N/R	From program authors: “Ongoing evaluation including DREEM, feedback through the consumer advisory council, and ongoing feedback provided by consumers, families and friends”
Rotary mental health awareness forums [64]	Post program feedback forms	Attendees at the forums	6548	N/R	Perceptions of good understanding of mental illness increased from 63 to 76% following the forums 64% of attendees had a good to very good awareness of what can be done to reduce the stigma of mental illness following the forums
SPEAK UP! Stay ChatTY [56, 57]	Post-session feedback is collected from participants from the Schools Program, Sports Program, Community Presentation and Mitch’s lived experience story. Pre-post data (not linked) is also available for Schools Program	Athletes from sporting clubs in Tasmania (Sports program). Students, teachers, parents from participating schools (Schools Program)	1239 (Sports program). Approx 1750 students (Schools Program)	Perceived knowledge and attitudes	Sports Program: Before the session, 818 (66%) athletes reported they knew ‘a bit’ about mental health, whereas after the session, 896 (72%) athletes stated they now know ‘a lot’. Likewise, before the session 673 (54%) athletes reported they knew ‘a bit’ about stigmatising signs of mental illness, however, after the session 869 (70%) athletes knew ‘a lot’ about stigmatising signs of mental illness Schools Program: Following the session, a majority (91.5%) felt more comfortable talking about mental health. There were also increases in perceived knowledge about mental health pre to post (A bit or a lot 81.6% to 97.0%) and perceived recognition of the signs of mental illness (A bit or a lot 63.0% to 96.6%)
The Dax Centre—Exhibition Program [61]	Post-feedback only	Exhibition visitors (86.4% were 16—17 year-old school students)	10,000	Response card with three statements with Likert scale response (Agree to Disagree) and brief written comments on any aspect of the person’s visit	Over 90% of respondents agreed that the exhibition helped them [1] gain a better understanding of mental illness, [2] gain a more sympathetic understanding of the suffering of people with mental illness; and [3] appreciate the ability and creativity of people with mental illness. These results were supported by the written feedback
The Station [31]	Qualitative interviews	Staff and members of a consumer-driven community mental health service	25	Interviews focused on The Station’s role in assisting recovery from mental illness, the limitations and strengths of the program, and relationships with the mental health system	Consumers reported feeling accepted and nurtured which increased feelings of empowerment and led to a greater belief in oneself from participating in the Station’s activities. Carers, consumers and volunteers all reported similarly of the positive impact of The Station on their lives. People who volunteer at The Station gain a sense of community and family, ‘time out’ and an opportunity to learn new skills and meet new people

*N/R* Not Reported

Programs targeted the following audiences:

#### Health professionals, health professional students, emergency workers

Our search identified seven programs that target health professionals, health professional students, or emergency workers. These varied in their approach but often included a focus on the potential for recovery, to counterbalance health professionals’ frequent contact with people when they are most unwell. Two programs target nursing students with contact interventions. One of these, *Recovery Camp*, is a nursing placement designed to facilitate contact between nursing students and people with lived experience outside an acute setting, where recovery is a focus. The program has run since 2013 and is funded by universities who pay for the placement by students. Two controlled trials found reduced anxiety about mental illness, negative stereotyping, and desire for social distance after the placement compared with traditional nursing placements. A second program, *Recovery for Mental Health Nursing Practice*, is taught by an academic with lived experience and also focuses on recovery concepts. Two controlled trials found improvements in some attitudes compared to a traditional mental health nursing subject. Pharmacy students are targeted by the *Remind Training and Education program*, which involves trained mental health consumers participating in pharmacy tutorials as educators. This program has run since 2010 and has reached 2,500 students at the University of Sydney. Evaluations in a controlled trial and an uncontrolled trial found reductions in stigma after the program and up to 12 months later. Of note, we identified one other program targeted to health students in a research study, but it is no longer running. This was a contact intervention for final year medical students to reduce stigma against people with schizophrenia as part of 6 week psychiatry rotation (see Additional file 1: Table S3).

Two programs target health professionals with education interventions. The *Collaborative Recovery Training Program* trains professionals in recovery concepts and is offered by the University of Wollongong. An uncontrolled trial found improved attitudes to consumers’ recovery possibilities after the training. The *Managing Mental Health Emergencies* short course trains rural and remote generalists how to respectfully and effectively manage mental health emergency care. An evaluation found better skills identifying psychosis and improved attitudes towards mental health clients. A third program, no longer running, focused on improving employment outcomes for consumers by funding Vocation, Education, Training and Employment Coordinators within mental health services (see Additional file 1: Table S2). An evaluation found an improvement in clinicians’ attitudes towards consumer capability of full-time, open employment.

Finally, *Mental Health Intervention Team training* is delivered to police officers and emergency service communication officers. The training is offered across an intensive 4-day program or 1-day training course. It teaches how to respond effectively during mental health emergencies with education and contact components. It has operated for more than 10 years in the NSW Police Force and Queensland Police Service. While an evaluation of a brief 2-h version for communications officers found no impact on stigma, a second controlled trial evaluating the full training package showed positive effects. Police officers reported increased confidence and understanding of how to deal with jobs involving individuals with a mental health problem or a drug induced psychosis.

#### People with mental illness

Eight programs target people with a mental illness (see Table 2). Most of these focus on reducing self-stigma, but some programs additionally aim to reduce public stigma through consumer participation in the community (i.e. contact). For example, *The Station* and *TasRec* both offer recreation programs where consumers engage with community members in a variety of activities. *The Station* aims to increase social connections and skills for living in people with a mental illness. It has operated since 1998 in South Australia and receives funding from a variety of sources. Interviews with participants found it increased feelings of empowerment and led to a greater belief in oneself. Similarly, *TasRec* provides recreation activities to help build skills, increase confidence, and reduce isolation. It has operated for more than 5 years in Tasmania by the Richmond Fellowship Tasmania and receives Commonwealth government funding. The Richmond Fellowship Tasmania also runs another program—*Residential Accommodatio*n, for people with mental illness. The service provides support to tackle stigma, access services, build social networks, and reach greater independence.

Two programs provide the opportunity for people with a mental illness to meet and support each other. The *Hearing Voices* group is a monthly/fortnightly peer support group for people with schizophrenia, who share stories and coping strategies on living with voices. It is offered in Victoria by Uniting Prahran. *The BPD Community Information Nights* are a forum for sharing information and support for people with Borderline Personality Disorder. They aim to address stigma and discrimination by focusing on hope and optimism about recovery. They are held three times a year in Victoria, supported by volunteers.

*My Recovery* is a peer-led education program for people living with mental illness offered in Darwin by Northern Territory Mental Health Coalition. The program aims to support recovery and provide a vocational pathway to people with lived experience. It is facilitated by peers and consists of nine weekly sessions that cover education topics such as stigma and discrimination, advocacy, recovery and skills training in communication, personalised recovery planning and goal setting.

A different sort of contact intervention is offered by *Compeer (The Friendship Program)*. Community volunteers and people with a mental illness are matched and meet regularly to develop friendships. The ACT branch of this international program has operated since 2009 with 253 participants. An evaluation found lower levels of stigma in volunteers with stronger relationships with their matches and that stigma was not related to the length of the relationship/contact.

Finally, *Being Herd* by batyr is a workshop for young people with mental illness who are trained how to share their stories to reduce stigma. This 2-day workshop has trained more than 700 people but has not been evaluated for its impact on stigma.

#### Families of people with mental illness

Three programs target families of people with mental illness (see Table 3). These include psychoeducation elements to increase understanding of mental illness and how to cope, and as such, may reduce self-stigma and stigma towards their family member, even though this may not be an explicit aim. The *BPD Community Family and Friends Group* provides support and psychoeducation. The group meets monthly and has operated in Victoria since 2015 on a volunteer basis. The *Journey to Recovery* is offered by St Vincent’s Mental Health Service in Victoria and has run since 2009. It is a group psychoeducation program for families and friends of people experiencing early psychosis to assist coping and reduce isolation. An outpatient version runs for 5 × 2-h sessions and an inpatient version is a single session. Two uncontrolled trials found improved knowledge of psychosis and recovery and reduced feelings of isolation in participants. A third program, *Kookaburra Kids Camps and Activity Days*, targets children of people with a mental illness. The program offers therapeutic recreation camps and activities in most states of Australia. Operating for more than 10 years, it has reached more than 3,000 people. Funding is from government, donations and corporate sponsorships.

#### Members of the general population

The most frequent target of anti-stigma programs was the general population, as we identified 11 programs of this type (see Table 4). Eight of these were training programs delivered in organisations such as schools, universities or workplaces. All programs focus on non-specific mental illness or mental illness including schizophrenia, psychosis, personality disorder, or bipolar disorder, rather than these disorders specifically. These programs are typically quite short, such as around 60 min in length. The exception is *Mental Health First Aid* training, which is at least 12 h in length. Six programs include both contact and education elements, one includes only contact and one includes only education.

Three programs have been established for more than ten years and have had a wide reach: *Mental Health 101*, *Mental Health First Aid* training, and SANE Australia’s *Peer Ambassador Program*. Mental Illness Education ACT (MIEACT) has run *Mental Health 101* courses for youth and adults in the ACT since 1993 with 8,000 people trained each year. These are 60-min workshops delivering contact and education to schools or workplaces. Consumer educators are guided by the DoNOHarm safe story-telling framework. A controlled trial of Mental Health 101 Youth found increased knowledge about mental illness and reduced stigma after the training. Interviews with the consumer educators showed that participating in the program had an effect on self-stigma but there was also a fear of being stigmatised as a result of presenting.

*Mental Health First Aid* (MHFA) training was established in 2000 and has trained 800,000 people across Australia. Training focuses on how to support a person developing a mental health problem or crisis and includes contact, education, and (optionally) a hallucination simulation activity. Training is delivered by accredited instructors who choose where to offer the course, such as workplaces, universities, and other organisations. To maintain program fidelity, accredited instructors are required to regularly deliver MHFA courses and undertake continuing professional development. MHFA has been rigorously evaluated in Australia and internationally since its inception with 3 meta-analyses, 16 RCTs, 7 controlled trials, and a number of uncontrolled trials. Meta-analyses show the program leads to a reduction in stigmatising attitudes after training and up to six months later [10]. Of note, the course has been evaluated in several culturally and linguistic diverse populations in Australia, including Vietnamese, Chinese, ‘multicultural’ communities, and Chinese international students, with positive effects on stigma. It has also been evaluated with health professional students, including nursing students and pharmacy students.

SANE Australia’s *Peer Ambassador Program* also involves presentations in workplaces and community settings across Australia by people with lived experience. Ambassadors receive training and support to share their personal experiences and also contribute to advocacy projects. This is a long-running program which currently supports 110 Peer Ambassadors, with more than 1000 trained since 1986. However, the program has not been evaluated for its impact on stigma.

Although only operating for 5–10 years, *batyr* has had already had a significant reach. Batyr run three programs—for schools (*batyr@school*), universities (*batyr@uni*) and workplaces (*batyr@work*). Sessions last 60–90 min and include contact from two people with lived experience as well as education about mental illness. Lived experience speakers are trained in the *Being Herd* workshop described above. Batyr programs have been delivered to more than 220,000 people and the batyr@school program has been evaluated with an RCT. This evaluation has not been published in the peer-reviewed literature but the authors report that the program reduced stigma towards mental health issues and this lasted for at least 3 months after the program.

Other similar awareness training programs delivered in organisations around Australia include *SPEAK UP! Stay ChaTY*, *LIVINGWell*, *Mental Health Awareness* by Mental Health Partners, and *Staff Wellbeing Workshop* by Chess Connect (delivered to workplaces in NSW only). *SPEAK UP! Stay ChaTY* has been evaluated and found participants reported being more comfortable in talking about mental health after the training. The other programs provided no information about evaluations.

Australian Rotary Health and Rotary Clubs hold *Mental Health Awareness Forums* in communities around Australia. These usually involve a consumer, a carer, and a mental health professional as speakers on mental health. Since 2000, about 5,000 people have participated in these forums. The program was evaluated with post feedback surveys and found improvements in perceptions of knowledge and what can be done to reduce stigma.

The Dax Centre in Victoria offers two complementary anti-stigma initiatives that are different to the programs described above—the *Exhibition Program* and the *Education Program (Mindfields)*. These are based around the exhibition of art by people with mental illness. The Exhibition Program educates the general public via the biographies of the artists. It has operated for more than 10 years and has received more than 24,000 visitors. Feedback forms from visitors show that most agree the exhibitions increase sympathy, understanding of mental illness, and appreciation of the creative ability of people with mental illness. The Education program is delivered to secondary and tertiary students and is a structured program including a tour of the exhibition, education and contact with people with lived experience. Another one-off program has also used art by people with lived experience to reduce self-stigma and stigma in the community (see Additional file 1: Table S3). Art created by young people attending Headspace in regional NSW was exhibited in commercial retail outlets and local community centres. Interviews with retail staff involved in the exhibition reported that the program had brought mental illness out into the open and increased empathic understanding of others’ emotional experiences.

Also of note is a one-off program that was run in Sydney’s Macedonian community to reduce stigma towards people with schizophrenia (see Additional file 1: Table S2). *Fear and Shame* was a theatre play about a Macedonian family with a son with schizophrenia. This was a culturally appropriate approach that reached about 1,600 people in the community over six months of staging. An uncontrolled trial of its impact found improved attitudes towards mental illness and a greater willingness to disclose and seek help from health services.

### Online resources accessible to the public

The search identified 19 online resources with a focus on reducing stigma towards mental illness that were designed and delivered by Australian organisations (see Table 6). These organisations provided a range of services and were not exclusively focused on running anti-stigma programs. Most organisations were not-for-profit or community sector (73%), with the remainder government (13%) and private (7%). Online resources were publicly and freely available by organisations via their websites or their content was uploaded to popular online streaming services such as Apple, Facebook and YouTube.

**Table 6:** Online resources accessible to the public

Online resource name	Organisation	Type of mental illness	Year	Target audience	Online resource description	Anti-stigma component	Lived experience involvement	Reach	Funding	Level of evidence
All in the Mind with Lynne Malcolm [65–71]	ABC	Range of disorders including bipolar disorder, borderline personality disorder, psychosis, and schizophrenia	2006 -	General population	A radio program that uses stories to explore the mind, brain, and behaviour. Several episodes have focused on mental illness and featured stories from people with lived experiences, as well as information from experts about treatments and recovery	Contact: Features stories from consumers and carers about their lived experiences and recovery Education: Featured guests include health educators and professionals who provide expert information and opinions about presentation, treatment and recovery	Consumers and carers with lived experiences are featured guests	N/R	Commonwealth gov	1
Bipolar Caregivers [72]	Private (Lesley Berk)	Bipolar disorder	2010	Carers of people with a mental illness	A website with online information for caregivers of people with bipolar disorder	Education: Evidence-based information and suggestions for caregivers about bipolar, its treatment and management, helping someone with bipolar, carer self-care, dealing with stigma or discrimination, and assisting someone dealing with stigma	Expert caregivers and people with bipolar disorder were consulted and informed evidence-based information featured on the website	N/R	NHMRC PhD scholarship funded	1
BPD Webinar Series [73]	Australian BPD Foundation, Spectrum, MHPN	Borderline personality disorder	2017	Health professionals	A six-part webinar series that features an expert panel of consumers, carers, and health professionals discussing their knowledge and experiences of borderline personality disorder and related topics	Contact: Panel members with lived experience provides knowledge and insight Education: Topics discussed by expert panel include information about BPD, treatment principles, evidence-based treatments and access, BPD in youth and early intervention, management of self-injury and suicidality, and management of BPD in Mental Health Services in Primary, Public, and Private Sectors	Consumers and carers with lived experiences are featured on expert panel	N/R	Commonwealth gov	2
Consumers and Carers as Educators [74]	Lived Experience Australia	Mental illness (non-specific)	2015	Health professionals	Several online training modules to inform and support health professionals	Contact: Features videos of interviews with consumers and carers Education: Features modules provide practical guidance on how to involve consumers and carers in meaningful ways into patient centred care models	Organisation comprises of people with lived experience as consumers and carers	N/R	Some funding from Mind Australia and RANZCP SA Trainees	1
Earshot [75]	ABC	Bipolar disorder	2015	General population	A radio program that presents a diverse selection of documentaries from intimate portraits to contemporary issues	Contact. One episode in the program features three people share their personal stories of bipolar disorder and bipolar mania to raise awareness	People with LE share their stories	N/R	Commonwealth gov	1
The Feed [76, 77]	SBS	Psychosis	2018	General population	A news, current affairs, and satire television series featuring two episodes interviewing people with lived experiences	Contact: One episode features a woman sharing her experience of post-natal psychosis to raises awareness of the stigma mothers with mental illness experience. Another episode features Osher Günsberg sharing his mental health experiences	People with LE share their stories	1,719,000 + views on Facebook 25,639 views on Youtube	Commonwealth gov	1
‘Let’s Talk’ podcast series [78]	Centre for Rural & Remote Mental Health	Severe mental illness including bipolar disorder, borderline personality disorder, and schizophrenia	2017	General population, people with lived experiences, rural and remote populations	A podcast series about mental health in rural and regional Australia	Contact and education: One episode has a focus on low prevalence mental illnesses featuring input from a person with lived experience and two professionals. It specifically addresses misconceptions, service provision in rural and remote Australia, treatment, and community engagement	The podcast was produced by a person with LE	N/R	University of Newcastle’s Centre for Rural and Remote Mental Health	1
Postnatal psychosis recovery stories [79]	PANDA	Postnatal psychosis	N/R	General population	A website featuring people’s stories about their lived experience of mental illness	Contact: Features online stories of mother’s lived experiences of postnatal psychosis including onset, treatment, management and recovery. Stories encourage knowledge and awareness of the potential for recovery	People with LE share their personal stories	N/R	N/R	1
Project Air Strategy website [80, 81]	Project Air Strategy	Personality disorders	N/R	Health professionals and people with lived experiences	A website with text and video stories of people’s lived experiences of personality disorders	Contact. Online text entries and videos featuring people’s stories of living with personality disorders including their day-to-day including parenting, diagnosis, self-help, treatment and recovery journeys Education: Features videos of health professionals and educators discussing treatment and stigma	People with LE share their stories	Personal stories amassed 2,881 views on Youtube	N/R	1
Recovery stories [82]	Neami National	Mental illness (non-specific)	N/R	General population	A website featuring people’s stories about their mental health journeys, including help-seeking and recovery	Contact: Features six people’s stories of recovery, including their positive experiences of support and formal service use	People with LE share their personal stories	N/R	N/R	1
SANE Forums [83, 84]	SANE Australia	Complex mental illness including bipolar disorder, psychotic illness, and bipolar disorder	2014	People with lived experiences (e.g., consumers, families, and carers)	Online peer-to-peer support for people living with mental illness and for carers	Contact: Provides a supportive online environment free of stigma for people to exchanges personal stories, seek opinions and similar experiences, discuss information and advice	People with LE respond to posts by others, provide active, helpful peer support to one another. Videos promoting SANE forums delivered by people with LE	N/R	Commonwealth gov	2
Say no to stigma! [85–89]	SANE Australia	Mental illness (non-specific)	2013	General population	A Youtube video campaign featuring people with lived experiences share their insight to tackle stigma in the general population	Contact: Videos feature people with lived experiences explain the impact of stigma and prejudice on their lives	People with LE share their insight	65,294 views on Youtube	Australian government, Department of Health and Ageing’s National Suicide Prevention Program	1
Social anxiety, stigma and early psychosis webinar [90]	Orygen	Psychosis, social anxiety	2017	Health professionals	An online training resource that informs clinicians, who work with young people, about social anxiety, stigma and early psychosis	Education: Features research evidence on service users’ perspectives of stigma, and explores the relationship between social anxiety, paranoid symptoms, negative beliefs about the self and the experience of shame. It also features research on treatment approaches for young people with social anxiety and early psychosis	N/R	N/R	N/R	1
Thriving Communities [91]	SANE Australia	Mental illness (non-specific)	2016	General population, people with lived experiences and rural and remote populations	A 14-week television, radio and online campaign to raise awareness of the benefits of online peer support and social connection for people affected by complex mental illness	Contact: Campaign features nine real-life stories of people affected by complex mental illness, including stories of living with bipolar, schizophrenia, and borderline personality disorder	People with LE share their personal stories	Reached 155 locations nationally, online stories amassed 66,938 views on Youtube	Commonwealth gov	2
Voices Vic Unplugged [92]	Uniting Prahran	Schizophrenia	2014	General population, people with lived experiences	A series of short films produced to reduce the extreme stigma experienced by people who hear voices	Contact: Videos feature real and personal stories from people about their experiences of hearing voices, and support, management and recovery	Peer-run campaign, videos directed by people with LE	7,317 views on Youtube	N/R	1
Website [93]	Australian Genetics of Bipolar Disorder Study	Bipolar disorder	2018	People with a mental illness	A website featuring people’s stories about their experiences of bipolar disorder	Contact: Features people’s stories of living with bipolar disorder, including treatment, management and recovery	People with LE share their personal stories	N/R	N/R	1
Website [94]	Borderline in the ACT	Borderline personality disorder	2017	General population, people with lived experiences, professionals (social service and welfare, health and emergency service), health professional students	A website to assist people with lived experiences and services providers find local services in the ACT and surrounding area. It also features evidence-based information	Contact: Features videos of people sharing their experiences of living with and managing BPD symptoms in their daily lives. Such videos highlight the complexities of BPD and stigma of BPD in the general population Education: Features support and local services information to help people assist those with BPD, and myth-busting to reduce stigma and foster empathy towards people with BPD	People with lived experiences were consulted on the design of the website, provide feedback on content and suggestions on supports and resources	10,000 + website visits	ACT health fund	2
#WeSpeakUp Campaign [95]	Consumers of Mental Health WA and Neami National collaboration	Mental illness (non-specific)	2019	General population	A series of videos featuring 13 West Australians with a lived experience of challenges with mental health and suicide	Contact: Videos feature people with LE share their stories of recognition, recovery and wellbeing to increase awareness of mental health issues and reduce stigma	People with LE share their stories	2,252 views on Youtube	Neami National	1
You Can’t Ask That [96]	ABC	Schizophrenia	2018	General population	A documentary program in which each episode asks controversial questions, sourced from the public, to a minority Australian population	Contact: One episode features eight Australians offer insight and break down stereotypes about living with schizophrenia	People with LE share their insight	65,750 views on Facebook	Commonwealth gov	1

1 = No evaluation evidence, 2 = Post survey feedback or qualitative interviews, 3 = One or more uncontrolled trials or repeated cross-sectional surveys, 4 = One or more controlled trials, 5 = One or more randomised controlled trials

*LE* Lived Experience, *N/R* Not Reported

Most online resources focused on reducing stigma towards non-specific mental illness (26%) and psychosis or schizophrenia (26%), with the remainder focused on ‘complex’ mental illness (16%), bipolar disorder (16%) and personality disorders, primarily borderline personality disorder (16%). The majority of online resources (63%) took a whole of population approach and used text, audio and video content to reduce self-stigma and public stigma. A minority of online resources identified additional targets to the general public; notably, ‘*Let’s Talk’*, a podcast series that also targets rural and remote populations and the *#WeSpeakUp* campaign, which features a diverse cross-section of people with lived experience, including members of the Aboriginal, LGBTIQ, and CALD communities. The remainder of online resources targeted specific groups such as health professionals (16%) and people with lived experiences (21%). The search identified three online resources for health professionals. These were primarily education based and focused on upskilling clinicians through online training modules and webinars.

Most online resources (84%) were contact-based or involved an element of contact, such as via online videos. Contact involved people with lived experience sharing their insight and stories, except for two that involved people with lived experience as experts in online training for health professionals (e.g., *BPD Webinar*, *Consumer and Carers as Educators*), and one that involved peer-to-peer support (e.g., *SANE Forums*). The remaining online resources were education-based or included an educational component.

People with lived experience were usually involved in delivering the content of online resources (89%). Most online resources did not report whether people with lived experience were involved in the design of the resources.

Online resources with notable reach were *SANE Forums* and specific episodes from television programs produced by national broadcasters—*The Feed* (SBS) and *You Can’t Ask That* (ABC). SANE Forums are an online peer-support community used by people living with mental illness and by family and other carers from around Australia. The forums provide a safe, supportive and stigma-free environment for users to build stronger connections with others affected by complex mental illnesses. Seventy-five partner organisations syndicate the Forums on their own websites. In the past 12 months the SANE forums were accessed by 35,000 Australians and gained 4,400 new members. Two episodes of *The Feed* feature interviews of people sharing their experiences of psychosis, treatment and recovery. These episodes have amassed more than one million views on Facebook. One episode of *You Can’t Ask That* features eight Australians providing insight into living with schizophrenia and addressing misconceptions of the public. This episode has amassed 65,750 views on Facebook.

The level of evidence for most online resources was low. No evaluation evidence was reported for the majority (79%). Of the remaining, evaluations were in the form of usage data, post-feedback surveys, or qualitative interviews only, and did not report on effects on stigma. Notably, an evaluation conducted on users of SANE Forums identified its value as a supportive online environment free of stigma, where people felt understood by others who had shared similar experiences.

### Awareness campaigns

We identified eight community campaigns in Australia with a focus on reducing stigma towards mental illness (see Table 7). These are mainly campaigns held annually that last for one day, one week, or one month. Apart from *BPD Awareness Week* and *Schizophrenia Awareness Week*, all campaigns focus on non-specific mental illness. All but two are conducted Australia-wide. Three campaigns have existed for more than ten years, two for 5–10 years, two for 2–5 years, and one was unclear. Six campaigns comprise multiple events, activities, promotional material and online/social media activity. There is a strong lived experience involvement in these campaigns, across their design, organisation, and delivery. Many of the events held as part of these campaigns include some form of contact between the public and people with a mental illness, whether that is via face-to-face stories or online video stories. Where reported, these campaigns have a wide reach from tens of thousands to hundreds of thousands each year. Only one campaign, *World Mental Health Day*, provided information from an evaluation. This was a feedback survey assessing satisfaction with campaign materials and willingness to participate in future, rather than assessing impact on stigma.

**Table 7:** Awareness campaigns

Campaign name	Organisation	Type of mental illness	Campaign activities	Anti-stigma messages/component	Lived experience involvement	Campaign duration	Where provided	Duration and reach	Funding	Level of evidencea
Mental Health Month	WayAhead—Mental Health Association NSW	Mental illness (non-specific)	Aims to raise awareness of the importance of mental health and wellbeing. Activities include Mental Health Matters Awards, Campaign theme, Collateral, Small grants to organisations to undertake activity, Community engagement, Advertising, Social media, website	The grant program supports organisations to undertake activity—some of which is contact interventions Campaign includes online stories of people with LE Key messages include ensuring the variety of causes of mental ill health are communicated and not reliant on medical model descriptors, violence is rare, majority of people recover etc	Led by a person with LE, some reference committee members have LE. Grant recipients must involve people with LE in activities	1 month	NSW, metro, regional/rural, remote	More than 10 years, hundreds of thousands	State gov	Unclear
World Mental Health Day (Australia)	Mental Health Australia	Mental illness (non-specific)	Aims to raise public awareness of mental health issues. 54 partners promoted the 2019 WMHD Campaign including the provision of partner specific posters and collateral, as well as partner specific events. Campaign participants were asked to make a mental health promise on the virtual promise wall at www.1010.org.au as a pledge of support, as well as share this promise via social media channels	Do You See What I See? Challenges perceptions about mental and encourages everyone to look at mental health in a more positive light, in an effort to reduce stigma and make way for more people to seek help and support There was consumer engagement and storytelling at the major event for the 2019 WMHD campaign in Townsville	18 consumers and carers were involved in helping inform the 2019 WMHD campaign. LE involvement in the design of photos and imagery of campaign	1 day (October 10)	Australia-wide. Metro, regional/rural, remote	More than 10 years, 100,000 + annually	Commonwealth gov	2
Mental Health Week (Tasmania)	Mental Health Council of Tasmania	Mental illness (non-specific)	A statewide campaign with the aim of reducing stigma around mental illness in the Tasmanian community. A number of health promotion events and activities held around the state all united under the one theme which last year was: We All Have a Role to Play	Several events included personal stories via talks or recorded interviews from people with lived experience. Most were around building resilience and community compassion—what interventions/supports have helped people	Many people with LE deliver, organise or facilitate MHW events. Steering committee has people with LE and carers	1 week	TAS, metro, regional/rural	5–10 years, ~ 13,000 per year	State gov	1
Mental Health Week (Northern Territory)	Northern Territory Mental Health Coalition	Mental illness (non-specific)	A statewide campaign with the aim of raising awareness about mental health and reducing stigma. It involves health promotion and events coordinated in partnership with member organisations and other mental health services	Various activities that involve the community and include an artwork display and competition, awards, and public forums. At events, printed materials are displayed and staff are available to discuss programs and interventions	People with LE are involved in all events. E.g., LE speakers at events, creating artwork or award recipients	1 week	NT, metro, regional/rural	2–5 years, 1,000 +	State gov, contributions from organisations	1
Schizophrenia Awareness Week [97]	Mental Illness Fellowship of Australia	Psychosis, schizophrenia	A seven-day awareness campaign that runs during Mental Health Awareness month. Government, citizens, media, and NGOs provide activities and events	Designed to raise awareness about schizophrenia and psychosis	N/R	1 week	Australia-wide. Metro, regional/rural, remote	More than 10 years, N/R	N/R	1
BPD Awareness Week	Australian BPD Foundation	Borderline Personality Disorder	Presentations by people with lived experience, online videos, postcards, posters and social media messages	The campaign highlights facts taken directly from The Clinical Practice Guideline for the management of Borderline Personality Disorder, in an accessible way The campaign also uses the strong voice of lived experience— promoting recovery, positivity and hope	People with lived experience are involved in the design of the campaign and collateral, providing quotes and video clips as well as presenting at events	1 week	Australia-wide, metro, online	2–5 years, some online posts reached 189,433 with 18,959 engagements	Commonwealth gov, volunteer	2
Odd Socks Day [98]	Grow	Mental illness (non-specific)	An annual national mental health anti-stigma campaign that encourages all Australians to wear odd socks on the Friday before Mental Health Week and World Mental Health Day	To support and show people struggling with their mental health that they are not alone	N/R	1 day	Australia-wide. Metro, regional/rural, remote	5–10 years, ~ 500,000 in 2018	N/R	1
One Sock One Goal [99]	batyr	Mental illness (non-specific)	Encourages teams and individuals to wear bright coloured batyr socks	To start positive conversations around mental health, whilst visually promoting their willingness to smash the stigma	N/R	N/R	N/R	N/R	N/R	1

1 = No evaluation evidence, 2 = Post survey feedback or qualitative interviews, 3 = One or more uncontrolled trials or repeated cross-sectional surveys, 4 = One or more controlled trials, 5 = One or more randomised controlled trials

*LE* Lived Experience, *N/R* Not Reported

Two campaigns take a different approach to those above—Grow’s *Odd Socks Day* and *batyr’s One Sock One Goal*. Both involve wearing socks as a way of raising awareness about mental illness and showing support. Odd Socks Day occurs annually on the Friday before World Mental Health Day and One Sock One Goal is ongoing.

Although no longer running, the *Napranum Social and Emotional Wellbeing Week* was a week-long campaign run in the Cape York community of Napranum (see Table 2 in supplementary material). This was led by a local steering committee with support from Townsville mental health services. The week comprised a variety of activities to reduce the stigma of mental illness, including MHFA courses, live radio shows and concerts, community breakfasts, and consultations with school and community organisations. Campaign feedback was positive and that perceptions of mental illness as frightening had reduced.

### Advocacy programs

Five programs were classified as advocacy initiatives (see Table 8). All programs focus on non-specific mental illness or mental illness including schizophrenia, psychosis, personality disorder, or bipolar disorder. Being’s *Mental Health and Wellbeing Consumer Advisory Group*, and *Lived Experience Australia*, both broadly advocate for improved acceptance of people with mental illness through activities such as participation in committees and media releases. Two programs advocated for change in specific areas—insurance coverage (Beyond Blue’s *Insurance Discrimination Project*), and media reporting (SANE’s *StigmaWatch*). The *Stop Mental Illness Stigma Charter* advocates for organisations to reduce stigma by committing to 7 principles. To date, 72 organisations have signed and committed to the charter. This program has existed for 2–5 years and a feedback survey from participating organisations showed 84% said adopting the Charter had made a difference in their organisation.

**Table 8:** Advocacy programs

Program name	Organisation	Type of mental illness	Advocacy description	Anti-stigma component	Lived experience involvement	Where provided	Duration	Funding	Level of evidencea
-	Lived Experience Australia	Mental illness (non-specific)	Active participation in multiple high-level steering committees/working groups/submissions in the capacity as recognised people with a lived experience of mental illness	Aim to reduce stigma making us the face of mental illness so people can see that mental illness is a diagnosis not who we are. No different from any other diagnosis, and that we can lead normal lives and contribute to society in a meaningful way	Organisation comprises people with lived experience as consumers and carers	Australia-wide	2–5 years	Volunteer, some funding from Mind Aust and RANZCP SA Trainees fund	1
Mental Health and Wellbeing Consumer Advisory Group	Being	Mental illness (non-specific)	Being promotes consumer issues widely within the mental health sector as well as within the public arena. Uses media releases that are responses to current community interests that promote understanding and acceptance of people with mental health issues	Being seeks understandings from consumers regarding their experiences of care, treatment and recovery, and seeks these opportunities to acknowledge the impact of stigma and discrimination as a personal experience as well as at a systemic state-wide level	100% of employees have LE. Consultation sessions are also conducted with consumers	NSW	More than 10 years	State gov	1
StigmaWatch	SANE Australia	Mental illness (non-specific), Bipolar disorder, Personality disorders, Psychosis, Schizophrenia, suicide, Eating Disorders	Ensures media outlets report safely and accurately on mental ill-health and suicide. If media coverage is found to breach Mindframe media reporting guidelines, StigmaWatch will contact the media outlet involved with constructive feedback and advice. StigmaWatch also highlights good, quality media stories that feature positive media portrayals. If the media do not remedy problematic reporting, we may report to the Press Council or take public action (e.g. media statements, letters to the editor etc.)	Aims to reduce the frequency and impact of stigmatising reporting in the Australian media	StigmaWatch has a long history of involving people with lived experience in thinking about how to design and deliver the program	Australia-wide	More than 10 years	Commonwealth gov	1
Stop Mental Illness Stigma Charter [100]	Murray PHN	Mental illness (non-specific)	The Charter aims to encourage organisations to adopt the right behaviours and practices and build an environment where employees and customers feel supported and understood. Organisations sign a pledge certificate and are supported with resources to implement the Charter	The Charter contains 7 commitments to reduce stigma: We will be informed; We will listen; We will be mindful of our language; We will be inclusive; We will challenge the stereotypes; We will be supportive; We will promote recovery	The concept was developed by a person with a lived experience. It was co-designed with people who have severe and persistent mental illness with complex needs	Australia-wide	2–5 years	Murray PHN (Commonwealth gov)	2
Insurance discrimination project [101]	Beyond Blue	Mental illness (non-specific)	Aims to improve access to insurance products such as travel, life, income protection and total and permanent disability insurance for people who have experience or are currently living with a mental illness	Reduction of discrimination from the insurance industry due to mental illness	N/R	Australia-wide	N/R	N/R	1

1 = No evaluation evidence, 2 = Post survey feedback or qualitative interviews, 3 = One or more uncontrolled trials or repeated cross-sectional surveys, 4 = One or more controlled trials, 5 = One or more randomised controlled trials

*LE* Lived Experience, *N/R* Not Reported

## Discussion

This review aimed to identify and examine the effectiveness of existing Australian programs or initiatives that aim to reduce stigma and discrimination towards people with complex mental illness that is poorly understood in the community. The broader aim was to inform options for a national stigma and discrimination reduction strategy as part of implementation of the Fifth National Mental Health and Suicide Prevention Plan in Australia.

Our review found 61 programs or initiatives currently available in Australia that had a focus on reducing stigma. These took a variety of stigma-reduction approaches across face-to-face programs, online resources, awareness campaigns, and advocacy work. The primary target audience for these initiatives were professionals (health or emergency), people with mental illness, family or carers of people with mental illness, and members of the general population. Most commonly, particularly for programs with a general public audience, programs tended to focus on stigma towards people with non-specific mental illness rather than on particular diagnostic labels. For some programs there may only have been a small component on poorly understood mental illnesses (i.e. schizophrenia, psychosis, personality disorder, bipolar disorder) and it is unclear whether anti-stigma messaging is diluted for these illnesses.

Evidence for whether programs are effective in reducing stigma is generally lacking. Only half of the face-to-face programs had been evaluated to test whether they had an impact on stigma, with only two programs evaluated with a ‘gold standard’ randomised controlled trial design. Nevertheless, most evaluations suggested positive effects on stigmatising attitudes. We did not find strong evidence of effectiveness for the other types of programs or online resources, noting that some of these are difficult to evaluate effectively.

There was little overlap of the programs identified in this review with those found in a meta-analysis of randomised controlled trials of international anti-stigma programs [9]. Only two programs were conducted in Australia, *Mental Health First Aid* training and a research-only program [11]. Although international programs have not been adopted into the Australian context, it is worth highlighting that most of the international studies did not evaluate ‘named’ programs that could be easily implemented elsewhere.

### Strengths in Australian practice

The most well-developed area is Australian face-to-face programs involving education and contact with a person with mental illness. This reflects the findings of a meta-analysis of randomised trials which showed both education and contact interventions were effective in reducing stigma [9]. It would appear that both approaches are complementary, as education can correct myths and misunderstandings that underpin stereotypes, and lived experience stories about recovery have an emotional resonance that make the impact of mental illness more tangible. Additionally, consumers who share their story of lived experience often receive a benefit of reduced self-stigma related to increased confidence, sense of meaning and connection to similar consumer peers. Consumers who take on this role usually receive training and ongoing support, and this is fairly extensive in some programs (e.g. a 2-day workshop).

Another strength is that most programs or initiatives have significant input from people with lived experience. This input is into program design (e.g. through co-design or acting as project advisors) and program delivery, often covering facilitation or co-facilitation, not just in presenting lived experience stories.

There are several programs that are examples of best practice in Australia, due to being well-established or sustainable and showing reasonable evidence of effectiveness. For the general population these are Mental Illness Education ACT’s *Mental Health 101* programs and *Mental Health First Aid* training. Other best-practice programs are *Recovery Camp* and the *Remind Training and Education* program for health professional students, and the *Journey to Recovery* program for family members of people with mental illness.

### Weaknesses in Australian practice

Several weaknesses were identified in current Australian practice. It should be noted that these weaknesses should be considered in light of some potential review limitations. Despite every attempt to reach relevant Australian organisations with our survey, we may have missed some programs as the survey was only open in the busy December/January holiday season. Further caveats are described below where relevant. Notwithstanding these potential limitations, there were very few programs that targeted culturally and linguistically diverse communities, Aboriginal and Torres Strait Islander communities and LGBTIQ people. Two programs that were identified, a culturally-appropriate theatre show for the Macedonian community, and a Social and Emotional Wellbeing campaign for the community of Napranum, were one-off programs that did not appear to be currently available.

Programs for people with mental illness and their carers or family members are not widespread and are generally only available in certain locations, such as particular mental health services or recreation programs provided by not-for-profits. However, it is possible that we did not identify some relevant psychoeducation programs for carers if they did not allude to reducing stigma as a focus. Nevertheless, we could not identify any programs that explicitly focus on reducing self-stigma, such as via psychoeducation, cognitive restructuring or disclosure approaches (e.g. Honest Open Proud 12). Broadening the search to programs that focused on empowerment may have identified further relevant programs for people with mental illness.

While there are some examples of best-practice stigma reduction for health professional students (particularly nursing and pharmacy students), these are not widespread in all Australian education programs. There are also few programs focusing on stigma for health professionals once they are practicing, with the caveat that there may be some continuing professional development resources on stigma that we could not access. The one initiative for mental health professionals that showed improvements in attitudes related to people with mental illness’s capacity to work was not systematically implemented after being defunded.

Finally, we only identified one available program for primary school students, which was a program for children of parents with a mental illness.

### Policy implementation recommendations

This review has highlighted the need for extensive consultations with key stakeholder groups to inform options for a national approach to stigma reduction in Australia. In particular, these consultations could address several questions that were unable to be answered in this review and provide guidance on implementation issues. Our review found programs offered to the general population tend to focus on non-specific ‘mental illness’, rather than complex mental illnesses that are poorly understood in the community. Given stigma varies by mental health problem, there is some debate about the merits of taking a generalist ‘mental illness’ approach, versus one that focuses more on specific mental illnesses and the particular issues associated with them [3]. Internationally, England’s Time to Change anti-stigma program uses non-specific mental illness in its approach and has shown positive effects upon stigma. Yet the authors of an evaluation of the program acknowledge that this may be because the public’s concept of mental illness has widened to include milder issues such as stress and grief, which are less stigmatised [13]. Consultations with Australian program providers and people with lived experience could explore views on whether existing programs targeted to mental illness should have a greater focus on complex mental illness, whether specific programs should be developed to do this, or whether existing programs should be implemented more widely.

Our review found that effective anti-stigma programs for nursing and pharmacy students exist but are not widespread, and we did not identify any effective programs for medical students. Consultations with health professional peak bodies and education providers could explore the options for development of new programs or sustainable expansion of existing programs that have evidence of effectiveness. Similarly, consultations with health care providers and people with lived experience could consider how to more widely implement into mental health services effective group psychoeducation for carers of people with early psychosis.

Overall, our review identified very few programs that target culturally and linguistically diverse or Indigenous communities. Consultations with these key stakeholder groups could identify communities in which anti-stigma initiatives are a priority, as well as how to support these communities in designing the most culturally appropriate sustainable interventions. There is also a need to expand the reach of programs that focus on reducing self-stigma in people with mental illness, and to identify which policy and funding mechanisms are required to do so.

### Conclusions

This study identified areas of strength and weakness in current Australian practice for the reduction of stigma towards people with complex mental illness that is poorly understood in the community. Most programs have significant input from people with lived experience, and programs involving education and contact with a person with mental illness are a particular strength. Nevertheless, best-practice programs are not widely implemented, and we identified few programs targeting stigma for people with mental illness and their families, or for culturally and linguistically diverse communities, Aboriginal and Torres Strait Islander communities and LGBTIQ people. These findings can inform implementation of Australian National mental health policy, with the aim of reducing stigma and discrimination and ultimately supporting social inclusion and recovery.

## Supplementary Information

**Additional file 1.** Additional tables.

## Data Availability

The datasets used and/or analysed during the current study are available from the corresponding author on reasonable request.

## Abbreviations

ACT: Australian Capital Territory
BPD: Borderline Personality Disorder
CALD: Culturally and linguistically diverse
LE: Lived Experience
LGBTIQ: Lesbian, Gay, Bisexual, Trans and gender diverse, Intersex, Queer and questioning
MHFA: Mental Health First Aid
MHIT: Mental Health Intervention Team
MIEACT: Mental Illness Education ACT
N/R: Not Reported
NGO: Non-government organisations
NSW: New South Wales
OCD: Obsessive Compulsive Disorder
PTSD: Posttraumatic Stress Disorder
QLD: Queensland
RCT: Randomised controlled trial
SA: South Australia
TAS: Tasmania
VIC: Victoria
