# Correction to: Review of Australian initiatives to reduce stigma towards people with complex mental illness: what exists and what works?

**DOI:** 10.1186/s13033-021-00490-y

**Published:** 2021-08-05

**Authors:** Amy J. Morgan, Judith Wright, Nicola J. Reavley

**Affiliations:** Centre for Mental Health, Melbourne School of Population and Global Health, University of Melbourne, 207 Bouverie Street, Melbourne, VIC 3010 Australia

## Correction to: Int J Ment Health Syst (2021) 15:10 https://doi.org/10.1186/s13033-020-00423-1

In the original publication of the article [1], in Table 2, the ‘Organisation column’ for the row ‘My Recovery’ was published incorrectly. It should read as “Wellways Australia and Northern Territory Mental Health Coalition” instead of “Northern Territory Mental Health Coalition”.

Under the subheading “*People with mental illness*”, third paragraph, the first sentence was published incorrectly. The corrected sentence should read as “My Recovery is a peer-led education program developed by Wellways Australia for people living with mental illness and is offered in Darwin through an auspice arrangement with the Northern Territory Mental Health Coalition”.
