# Supplementary material for: Review of Australian initiatives to reduce stigma towards people with complex mental illness: what exists and what works?
Source: Int J Ment Health Syst. 2021 Jan 18;15:10. doi: 10.1186/s13033-020-00423-1 (PMC7814561; doi:10.1186/s13033-020-00423-1)
Supplement: Supplementary file 1 — Additional file 1. Additional tables. [file 13033_2020_423_MOESM1_ESM.docx]

Table S1. Search strategy for PubMed

| 1 | (stigma[TIAB] OR stigmatization[TIAB] OR stigmatisation[TIAB] OR anti-stigma[TIAB] OR "Social Stigma"[Mesh] OR "Social Discrimination"[Mesh] OR "Attitude"[Mesh] OR "Attitude of Health Personnel"[Mesh] OR "Prejudice"[Mesh] OR "Social Distance"[Mesh] OR Stereotyping[Mesh] OR Social Perception[Mesh] OR "Rejection (Psychology)"[Mesh] OR Shame[Mesh]) |
| --- | --- |
| 2 | (Mental Disorders[Mesh:NoExp] OR "Schizophrenia Spectrum and Other Psychotic Disorders"[Mesh] OR "Bipolar and Related Disorders"[Mesh] OR "Mentally Ill Persons"[Mesh] OR "Personality Disorders"[Mesh] OR schizophrenia[TIAB] OR psychosis[TIAB] OR psychotic[TIAB] OR bipolar[TIAB]) |
| 3 | ("Program Evaluation"[Mesh] OR "Health education"[Mesh] or "Patient education as topic"[Mesh] or teaching[Mesh] OR "Health promotion"[Mesh] or "Healthy people programs"[Mesh] OR "Program Development"[Mesh] OR "Inservice training"[Mesh] or "Staff development"[Mesh] OR intervention[TIAB] or film[TIAB] or video[TIAB] or campaign[TIAB] or initiative[TIAB] or awareness[TIAB] or program[TIAB] or training[TIAB] or workshop[TIAB] or intervene[TIAB] or seminar[TIAB] or curriculum[TIAB] or "booster session"[TIAB] or strategy[TIAB] or strategies[TIAB] or implement[TIAB] or course[TIAB] or symposium[TIAB] or symposia[TIAB] or coaching[TIAB] or mentoring[TIAB] or policy[TIAB] or policies[TIAB] or guideline[TIAB] or recommend[TIAB] or leaflet[TIAB]) |
| 4 | ((Australia[TIAB] OR Australian[TIAB]) OR Australia[AD]) |
| 5 | 1 AND 2 AND 3 AND 4 |
| 6 | ("Review" [Publication Type] OR "Cross-Sectional Studies"[Mesh] OR "Meta-Analysis" [Publication Type]) |
| 7 | 5 NOT 6 |
| 8 | Limit to Publications >= 2009 and Language = English |

Table S2. One-off or ceased programs

| Program name | Organisation | Type of mental illness | Target audience | Program description | Anti-stigma component | Facilitated by | Where provided | Duration and reach | Funding | Level of evidence |
| --- | --- | --- | --- | --- | --- | --- | --- | --- | --- | --- |
| Art-making and exhibition program (1, 2) | Headspace | Mental illness (non-specific) | Young people, general population | Art created by young consumers as part of an art group was exhibited in commercial retail outlets and local community centres. | Other: Art-making process to reduce self-stigma indirectly through improving agency, empowerment, and social connectedness in artists.  Public display of art to reduce stigma and social marginalization of young artists with mental illnesses by promoting positive views, understanding and acceptance in exhibition visitors. | Artists, Headspace and various commercial retail outlets | NSW,  Regional | Art displayed for 2 months for one study, N/R for the other, N/R | Private funding from artist facilitators, Australian College of Mental Health Nursing, Headspace | 2 |
| Bipolar Education Program (BEP) (3) | University of NSW | Bipolar disorder | People with lived experiences | An online eight module psychoeducation program for people with bipolar disorder. | Educational: Information on causes of bipolar disorder, support networks, diagnosis, treatments, and wellbeing plans. Included personal testimonies and advice from well-known people with the disorder and interviews with experts. | N/A | Online | Eight weeks, 273 | N/R. | 5 |
| Fear and Shame (4) | St. George Mental Health Service | Mental illness (non-specific) with a specific focus on schizophrenia | Australian Macedonian community | A theatre play creatively exploring mental illness and stigma in the Macedonian community. | A culturally appropriate strategy to reduce the stigma of mental illness and promote mental health in the Macedonian community. Uses key scenes to highlight the challenges experienced by a Macedonian family whose family member has schizophrenia, including help-seeking and negative reactions from friends and neighbours. | Australian Macedonian Theatre of Sydney | NSW,  metro | Staged over six months, 1,600 people | South Eastern Sydney and Illawarra Health (SESIH) Mental Health Service and Multicultural Health Unit, Australian Council, Hurstville City Council, Community Relations Commission. | 3 |
| N/R (5) | Ballan District Health and Care | Borderline personality disorder | Health professionals | Lectures delivered during staff developed at a health service to change clinicians’ attitudes toward deliberate self-harm behaviours in borderline personality disorder. | Both lectures included components on research-based information and clinical guidelines. One component differed lectures, with either a presentation on three case studies illustrating the use of self-harm as a coping mechanism from a cognitive behavioural or psychoanalytic perspective. | Psychologist | VIC | N/R,  43 | N/R | 4 |
| N/R (6) | St George Mental Health Service | Mental illness (non-specific) | Macedonian community, Macedonian-speaking hospital staff. | A multifaceted community education intervention to reduce stigma, improve health literacy and access to mental health care in the Macedonian community. | Educational: The community education session included specific key messages to address negative perceptions of mental illness and consequences of stigma. | N/R | NSW | N/R | Two Diversity Health Grants from the South Eastern Sydney Area Multicultural Health Unit | 2 |
| Napranum Social and Emotional Wellbeing Week (7) | Community Forensic Outreach Service (CFOS) and the Adolescent Forensic Mental Health Service (AFMHS) | Mental illness (non-specific) | Napranum community | A week-long outreach project aimed to reduce the stigma of mental illness and improve understanding of mental health and illness from a holistic ‘wellbeing’ perspective. In the Napranum community, and strengthen partnerships among service providers and government agencies through various community events. | Education: Two MHFA courses provided to community members and community health workers.  Community campaign: Professional consultation of project team members with staff at a school and various community-based centres (e.g., youth club, women’s shelter), and extensive networking between health agencies. Variety of community events (e.g. live radio show, concert, community breakfasts). | Variety of local agencies and Napranum Council. | QLD,  Remote (Cape York community of Napranum) | One week, N/R | A range of sources (not specified) | 2 |
| SIGNS campaign (8) | SANE | Mental illness (non-specific) | General population, friends and family members | A media campaign targeted towards friends or family as people being in the best position to recognise the early signs of mental illness in another person. | Encompassed various media sources (e.g., radio, TV, and press ads) designed to create further acceptance of mental illnesses as real, increase awareness of the possible symptoms, and encourage families of people with suspected mental illness to contact SANE for help. | N/A | Online, Australia wide | N/R | N/R | 1 |
| SMILE (Simple, Mental heath, Initiative in Learning and Education) (9) | Southern Cross University | Mental illness (non-specific) | Health professional students | A program providing further training and support for student learning in mental healthcare. Some topics covered in the program included psychosis, suicide prevention, families and carers in mental health, and the Mental Health Act. | Educational: Presentation of case study scenarios designed to stimulate discussion related to social stigma and mental illness, psychiatric practice and the future of mental healthcare. | Clinical educator | N/R | Started in 2010, N/R |  | 3 |
| There is Hope: Schizophrenia Awareness Program (10) | SANE Australia, Home and Away | Schizophrenia | General population with a focus on young people | A collaboration between SANE Australia and the television show Home and Away, to develop and produce a story line on schizophrenia. | Contact: One of the regular characters develops schizophrenia in a story run over 4 months reflecting an accurate and sympathetic portrayal of schizophrenia.  Campaign: Character’s image was used as a springboard national campaign on educational posters and pamphlets distributed via GPs, schools and clinics to raise additional awareness. | Home and Away | Australia wide | Episodes ran over a 4-month period in early 1999, N/R | Friends of SANE Australia and pharmaceutical company Eli Lilly and Company | 1 |
| Vocation, Education, Training and Employment (VETE) Coordinators (11) | Formerly South East Sydney Illawarra Area Health Service (SESI) | Bipolar disorder, Schizophrenia, Depression | Health professionals | Establishment of VETE Coordination positions in mental health services to improve employment outcomes for consumers by providing specialist, evidence-based VETE interventions as a routine part of rehabilitation service provision. | VETE Coordinators provide specialist consultation to the mental health service and establish links with organisations providing direct job seeking assistance. They also provide guidance, support, education and information to mental health service teams and local employment and educational organisations with the aim of establishing successful VETE pathways and partnerships. | Mental health professionals acting as VETE Coordinators | NSW | Started in 2007. | N/R | 3 |

1 = No evaluation evidence, 2 = Post survey feedback or qualitative interviews, 3 = One or more uncontrolled trials or repeated cross-sectional surveys, 4 = One or more controlled trials, 5 = One or more randomised controlled trials

LE = Lived Experience, N/R = Not Reported.

Table S3. Research studies

| Study authors (year) | Type of mental illness | Program description | Anti-stigma component(s) | Experimental design | Study sample and size | Measures | Outcomes |
| --- | --- | --- | --- | --- | --- | --- | --- |
| Galletly 2011 (12) | Schizophrenia | An intervention for final year medical students to reduce stigma against people with schizophrenia as part of six-week psychiatry rotation. | Contact: A 40-minute DVD narrative about a young university student who develops schizophrenia. Describes his illness well and ability to continue working and maintain a strong social network. He is engaging and likeable.  Simulating hallucination symptoms: Participants watch a 10-minute segment of a TV show in which a well-known presenter participated in a simulated hallucination workshop at a mental health conference. Then they listened to 45-minute simulated hallucinations via mp3 player (Australian voices) and completed tasks with another student. Voices did not include derogatory, suicidal or homicidal content. | Uncontrolled trial (pre/post) | Medical students (N=87) | Attitudes to Mental Illness Questionnaire | Stigmatising attitudes reduced pre-to-post (p<.001), particularly in students with more negative attitudes at baseline (bottom half). Those with average or better attitudes at baseline did not improve. Qualitative feedback showed that Students found the workshop useful and described a better understanding of the everyday difficulties of living with chronic psychotic symptoms. |
| Giacobbe, Stukas, & Farhall (2013) (13) | Schizophrenia | Two contact-based interventions to reduce stigma against people with schizophrenia. | Contact: Participants asked to imagine an interaction with an adult diagnosed with schizophrenia that was positive, relaxed and comfortable. Imagined interaction followed a script provided by researchers that involved an informal chat. Or, participants interacted with a confederate who said he had schizophrenia. | RCT (pre/post) | General university students (psychology). 97 (48, 49) | Dangerousness Scale (Link et al., 1987), Affect Scale (Penn et al., 1994), Social Distance Scale (Link et al., 1987, Penn et al., 1994) | Stigmatising attitudes and social distance reduced reduce pre-to-post (p<.01) for face-to-face and imagined contact. Negative affect reduced over time, but there was no difference between contact and control conditions. |
| Kenny (2016) (14) | Mental illness (non-specific) | One intervention was based on Acceptance and Commitment Therapy (ACT) and one was an education intervention. | Education intervention provided information about prevalence of mental illness, stigma and its impact. Interactive activities involving group discussion were included.  ACT intervention emphasised the way that language can create and maintain stigmatizing attitudes. Activities demonstrated link between stigmatizing attitudes and cognitive processes and the role of acceptance and nonjudgement. | RCT (2 active conditions) (pre/post) | University psychology students (n = 152 (ACT = 71, education = 81) | Prejudice towards People with Mental Illness. Comprises total score and 4 dimensions of fear/avoidance, malevolence, authoritarianism, and unpredictability | Overall prejudice reduced in both conditions, but the reduction was stronger in the ACT group (p<.001) than the education group (p=.023). All subscales improved except for malevolence, which worsened in both conditions. |
| Morrison (2009) (15) | Mental illness (non-specific) | Classroom activity to explore nursing students’ understandings of mental illness and mentally ill people. Involves a narrative approach to understand lived experience via an emphasis on talents, competencies and achievements, rather than problems. | Education: Students completed an assignment on how mental illness is represented in the popular media. Students shared their work in tutorials and reflected on the stories presented. | Qualitative | Nursing students, N/R | Discussion from tutorials on what they learned about mental illness | Students reported understanding more about how stigma is influenced by the media from a young age; that it was possible to reduce stigma as an individual; what it might be like for people with mental illness and their struggles to belong; that their assumptions about mental illness had been challenged; better understanding of what life might be like for a person with mental illness and their families, some of the causes of stigma and the public’s perceptions of mental illness. |
| Thorsteinsson (2019) (16) | Schizophrenia | An online educational video sourced from YouTube. | Educational video explained the symptoms and possible causes of schizophrenia, including both psychosocial and biological, and the stigma about schizophrenia, selected from YouTube. | RCT (pre/post) | General public (online, 66% from Australia), n = 260 (Intervention = 129, control = 131) | Personal stigma, perceived stigma, social distance and perceived discrimination. | Significant small reduction in personal stigma in the education condition for participants with a health background, but no change for those without a health background. There was a reduction in personal stigma in the no health background group in the control condition. |
| Ventieri, Clarke & Hay (2011) (17) | Mental illness (non-specific) | An educational intervention on mental illness delivered to Australian primary school children. | Interactive in nature, uses role-plays, games and activities. Curriculum divided into four parts: (1) general introduction to mental illness, (2) causes of mental illness, (3) possible treatments for people with mental illness, and (4) stigma and mental illness. General themes addressed in the fourth part were that the adverse impact of stigmatizing attitudes, negative language perpetuating mental illness stigma, and awareness around the words used to describe people with mental illness. | Controlled trial (pre/post, four month follow up) | Primary school students, n = 195 (Intervention = 69, control = 126) | Attitudes towards mental illness; Social distance scale | Significant increase in attitudes (‘Unkindliness’ (p<.001), ‘Benevolence’ (p=.001)) and knowledge (p<001), and reduction in desire for social distance (p<.001) for the intervention group compared with the control group at one-week follow up. This effect was maintained at four-month follow up (attitudes (‘Unkindliness’ (p<.001), ‘Benevolence’ (p=.012)), knowledge (p<.001), and social distance (p=.005)). |

1 = No evaluation evidence, 2 = Post survey feedback or qualitative interviews, 3 = One or more uncontrolled trials or repeated cross-sectional surveys, 4 = One or more controlled trials, 5 = One or more randomised controlled trials

LE = Lived Experience, N/R = Not Reported.

**References**

1. Gentle E, Linsley P, Hurley J. "Their story is a hard road to hoe": how art-making tackles stigma and builds well-being in young people living regionally. Journal of Public Mental Health. In press.

2. Hurley J, Linsley P, Rowe S, Fontanella F. Empathy at a distance: A qualitative study on the impact of publically-displayed art on observers. International Journal of Mental Health Nursing. 2014;23(5):419-26.

3. Proudfoot J, Parker G, Manicavasagar V, Hadzi-Pavlovic D, Whitton A, Nicholas J, et al. Effects of adjunctive peer support on perceptions of illness control and understanding in an online psychoeducation program for bipolar disorder: a randomised controlled trial. Journal of Affective Disorders. 2012;142(1-3):98-105.

4. Blignault I, Smith S, Woodland L, Ponzio V, Ristevski D, Kirov S. Fear and shame: using theatre to destigmatise mental illness in an Australian Macedonian community. Health Promotion Journal of Australia. 2010;21(2):120-6.

5. Treloar AJ. Effectiveness of education programs in changing clinicians' attitudes toward treating borderline personality disorder. Psychiatric Services. 2009;60(8):1128-31.

6. Blignault I, Woodland L, Ponzio V, Ristevski D, Kirov S. Using a multifaceted community intervention to reduce stigma about mental illness in an Australian Macedonian community. Health Promotion Journal of Australia. 2009;20(3):227-33.

7. Hartman D, Wallis G, Drahm M, Unwin R, Robinson D. The Napranum social and emotional wellbeing week. Australasian Psychiatry. 2009;17 Suppl 1:S88-91.

8. Harman G, Heath J. Australian country perspective: The work of beyondblue and SANE Australia. The stigma of mental illness - End of the story? Cham, Switzerland: Springer International Publishing; 2017. p. 289-315.

9. Ward LJ. SMILE: Simple, Mental Health, Initiative in Learning and Education. Education for Health. 2011;24(3):537.

10. Sartorius N. Compendium of programmes aiming to reduce stigma and discrimination because of schizophrenia or mental illness in general [Internet] 2002 [Available from: <https://www.openthedoors.com/english/media/vol_4.pdf>.

11. Sommer J, Lunt B, Rogers J, Poole R, Singham A. The impact of Vocational, Education, Training and Employment Coordinator positions on attitudes and practices in a NSW mental health service. Australasian Psychiatry. 2012;20(4):295-300.

12. Galletly C, Burton C. Improving medical student attitudes towards people with schizophrenia. Australian and New Zealand Journal of Psychiatry. 2011;45(6):473-6.

13. Giacobbe MR, Stukas AA, Farhall J. The effects of imagined versus actual contact with a person with a diagnosis of schizophrenia. Basic and Applied Social Psychology. 2013;35(3):265-71.

14. Kenny A, Bizumic B. Learn and ACT: Changing prejudice towards people with mental illness using stigma reduction interventions. Journal of Contextual Behavioral Science. 2016;5(3):178-85.

15. Morrison PA. Using an adapted reflecting team approach to learn about mental health and illness with general nursing students: an Australian example. International Journal of Mental Health Nursing. 2009;18(1):18-25.

16. Thorsteinsson EB, Bhullar N, Williams E, Loi NM. Schizophrenia literacy: the effects of an educational intervention on populations with and without prior health education. Journal of Mental Health. 2019;28(3):229-37.

17. Ventieri D, Clarke DM, Hay M. The effects of a school-based educational intervention on pre-adolescents' knowledge of and attitudes towards mental illness. Advances in School Mental Health Promotion. 2011;4(3):5-17.
